# Decreased Oxytocin Mediates PVN–CA2 and PVN–PrL in Sleep Deprivation-Induced Social Memory Deficits

**DOI:** 10.34133/research.1076

**Published:** 2026-02-06

**Authors:** Yanchao Liu, Yuchen Deng, Yang Gao, Bo Rao, Yuxin Wang, Yifei Zhang, Kebing Yi, Yufeng Cang, Haiyang Li, Linlin Bi, Haibo Xu

**Affiliations:** ^1^ Department of Radiology, Zhongnan Hospital of Wuhan University, Wuhan University, Wuhan 430071, China.; ^2^ Department of Clinical Research and Translational Medicine, The Third Affiliated Hospital of Zhengzhou University, Zhengzhou 450052, China.; ^3^ Department of Pathology, Taikang Medical School (School of Basic Medical Sciences), Wuhan University, Wuhan 430071, China.; ^4^ School of Chemistry and Molecular Science, Wuhan University, Wuhan 430071, PR China.; ^5^ Department of Radiology, The Third Affiliated Hospital of Zhengzhou University, Zhengzhou 450052, China.; ^6^ Center for Pathology and Molecular Diagnostics, Zhongnan Hospital of Wuhan University, Wuhan University, Wuhan 430071, China.; ^7^ Hubei Provincial Engineering Research Center of Multimodal Medical Imaging Technology and Clinical Application, Wuhan 430071, China.; ^8^ Wuhan Clinical Research and Development Center of Brain Resuscitation and Functional Imaging, Wuhan 430071, China.

## Abstract

While sleep disorders are a known correlate of social memory deficits, the underlying neurocircuitry and molecular mechanisms remain poorly understood. Using an oxytocin (OXT)-specific sensor imaging approach, we discovered that chronic sleep deprivation (SD) reduced OXT neuropeptide release in the hippocampal CA2 and prelimbic cortex (PrL), thereby disrupting social memory encoding and retrieval processes, respectively. Using fiber photometry recordings and in vitro electrophysiology, we identified the activity of the predominantly OXT-expressing neurons in the paraventricular hypothalamic nucleus (PVN^OXT^) were reduced following SD. Specific optogenetic activation of the PVN^OXT^–CA2 pathway during encoding phase or PVN^OXT^–PrL pathway during retrieval transiently restored SD-induced social memory deficits. Conversely, optogenetic high-frequency activation of PVN^OXT^ neurons enhanced the function of both PVN^OXT^–CA2 and PVN^OXT^–PrL pathways, promoting increased OXT release and providing sustained protection against SD-induced social memory deficits. These findings offer causal evidence that the PVN^OXT^–CA2 and PVN^OXT^–PrL pathways exert distinct modulatory roles in sleep-related social memory deficits and thereby nominate these pathways as precise targets for neuromodulation in sleep-related cognitive disorders.

## Introduction

Social memory, which is the capacity to identify familiar individuals and engage with unfamiliar conspecifics, is a core component of social cognition. Deficits in this domain are prevalent across a broad spectrum of psychiatric and neurological conditions [1,2], such as autism spectrum disorder (ASD), posttraumatic stress disorder (PTSD), and Alzheimer’s disease (AD). Many patients with these conditions also exhibit reduced sleep duration or disrupted sleep architecture [3–5]. Chronic sleep deprivation (SD) impairs social memory [6], but the circuit-level mechanisms underlying this effect remain largely unclear.

Within the hippocampus, pyramidal (PYR) neurons located in the CA2 subfield play a critical role in processing social novelty, facilitating the differentiation between new and previously encountered individuals [7,8]. Social memory is enhanced by activating the supramammillary nucleus–CA2 circuit [9] or vasopressinergic projections from the paraventricular hypothalamic nucleus (PVN) to CA2 during novel social encounters [10]. While the hippocampus supports encoding, the prefrontal cortex (PFC) is primarily involved in memory retrieval [11]. In particular, excitatory PYR neurons within the PFC have been shown to support the recollection of socially relevant experiences, and stimulation of PFC–nucleus accumbens projections can thereby support the retrieval of social memory for a previously encountered but apparently forgotten mouse [12].

The prelimbic cortex (PrL), a medial subdivision of the PFC, is notable for its high expression of oxytocin (OXT) receptors [13]. OXT, a neuropeptide pivotal to the regulation of affiliative, reproductive, and parental behaviors [14,15], exerts prosocial effects through its receptor (OXTR). OXTRs are abundantly expressed in CA2 and CA3 PYR neurons, and conditional deletion of OXTR in these regions impairs the persistence of long-term social recognition memory [16]. OXT signaling shapes neuronal excitability, firing patterns, and synaptic plasticity in CA2, processes essential for oscillation generation and social memory encoding [17]. Therefore, characterizing OXT signaling dynamics in the CA2 and PFC is essential for understanding how OXT modulates social memory circuits. The recent development of G-protein-coupled-receptor-based OXT sensors now permits high-sensitivity imaging of OXT release in both ex vivo and in vivo settings [18,19]. Despite this advanced tool, OXT dynamics during social exploration in the CA2 and PrL have not been investigated, leaving a critical gap in understanding the circuit-level mechanisms by which OXT regulates social memory. Given the pivotal role of the PVN, rich in OXT-positive neurons, in regulating social behaviors [20–22], we hypothesized that the PVN^OXT^–CA2 and PVN^OXT^–PrL pathways play distinct roles in encoding and retrieving social memory and that dysfunction in these pathways contributes to SD-induced deficits.

We used OXT-specific sensor imaging to dissect the spatiotemporal dynamics of OXT signaling in these circuits. We found that SD disrupted PVN^OXT^–CA2 and PVN^OXT^–PrL activity, impairing social memory encoding and retrieval, respectively.

Optogenetic activation of the PVN^OXT^–CA2 pathway during encoding phase or PVN^OXT^–PrL pathway during retrieval transiently restored SD-induced social memory deficits. Moreover, high-frequency stimulation of the predominantly OXT-expressing neurons in the PVN enhanced OXT release and strengthened circuit connectivity, offering sustained protection against memory impairments. This study provides causal evidence that the PVN^OXT^–CA2 and PVN^OXT^–PrL pathways exert distinct modulatory effects, offering novel insights into sleep-related social memory deficits and identifying potential therapeutic strategies and targets.

## Results

### Chronic SD induced lasting social memory impairment

**Fig. 1.**
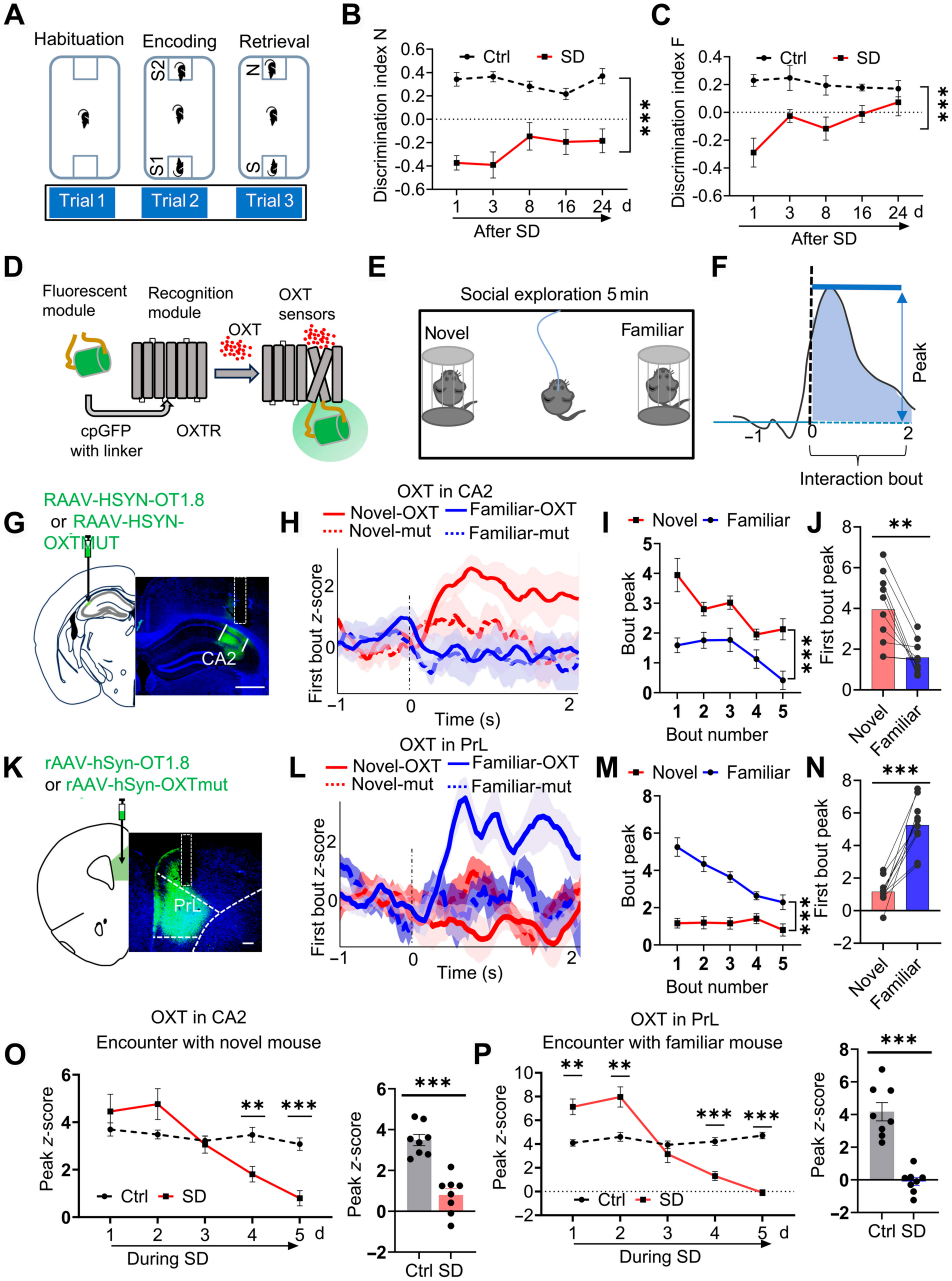
Chronic sleep disruption reduces OXT release in the CA2 and PrL and impairs social memory. (A) Schematic of the 2-choice social memory test comprises 3 phases: habituation, encoding, and retrieval. Initially, the test mouse was acclimated to the setup with 2 empty cages (trial 1: habituation). Subsequently, 2 novel stimulus mice (S1 and S2) were placed individually in separate cups, and the subject mouse was allowed to explore both for 5 min (trial 2: social memory encoding). To evaluate social memory retrieval, a third trial was conducted 30 min after the second. The subject mouse was placed back into the arena for 5 min, during which one previously encountered mouse (S1 or S2) was replaced by a novel mouse (N), and the other was retained as the familiar stimulus (S). Discrimination index N = (time spent exploring mouse N − time spent exploring mouse S) / (time spent exploring mouse N + time spent exploring mouse S). Discrimination index F = (time spent exploring mouse S1 or S2 during trial 2 − time spent exploring mouse S during trial 3) / (time spent exploring mouse S1 or S2 during trial 2 + time spent exploring mouse S during trial 3). Normal social memory is characterized by the following: (a) an increased exploration time of the novel mouse (N) compared to the familiar mouse S (S1 or S2) during trial 3, measured by the discrimination index N, and (b) a reduced exploration time of mouse S (S1 or S2) in trial 3 relative to trial 2, measured by the discrimination index F. (B) Sleep-deprived mice exhibited reduced exploration time with the novel mouse (N) and a lower discrimination index N through post-SD day 24. Two-way ANOVA analysis; *n* = 5 mice in control (Ctrl) group and *n* = 5 mice in SD group. (C) Sleep-deprived mice spent more time exploring the familiar mouse (S) and showed a lower discrimination index F through post-SD day 24. Two-way ANOVA analysis; *n* = 5 mice in Ctrl group and *n* = 5 mice in SD group. (D) Diagram of the action principle of OXT-specific sensors-GRAB_OXT1.8_ exhibits fluorescence changes upon OXT binding to its receptor. (E) The subject mice received GRAB_OXT1.8_ sensor, and a social interaction test was conducted alongside simultaneous fiber photometry. (F) The peak *z*-score and average AUC for the first bout of each interaction category were analyzed, as described in the literature [6]. OXT traces during the first interaction bouts with the novel and familiar mouse were aligned to the bout onset (time of 0 s). (G to J) OXT or OXTmut signaling dynamics in the PrL during exploration of familiar and novel mice. OXT fluorescent signals in CA2 increased during the first interaction bouts with novel mice, while no marked change is observed in presence of a familiar mouse. The OXTmut signaling did not show any noticeable changes. OXT, *n* = 10 mice; OXTmut, *n* = 6 mice. Scale bar, 500 μm. (G) Schematic of rAAV-hSyn-OT1.8 or rAAV-hSyn-OTmut virus injection into the CA2 region and a representative post hoc histological image of the recording site in CA2. (H) OXT and OXTmut traces in CA2 during the first interaction bouts with novel mouse aligned to the bout onset (time of 0 s). Perievent plots display averaged fluorescence, with curves and shaded regions indicating the means ± SEM. Paired *t* test; *n* = 10 mice per group. (I) The peak *z*-score of OXT signals in the CA2 was calculated from the first 5 bouts of interactions with novel and familiar mice. Two-way ANOVA analysis. (J) The peak of OXT signaling in the CA2 was calculated for the first interaction bouts of each category. (K to N) Dynamics of OXT or OXTmut signaling in the PrL during social interactions with familiar versus novel mice. Experimental structure and analysis were consistent with those described in (G) to (J). OXT fluorescence signals in the PrL markedly increased during initial interaction bouts with familiar mice, whereas no marked change was observed during encounters with novel mice. The OXTmut signaling did not show any noticeable changes. OXT, *n* = 10 mice; OXTmut, *n* = 6 mice. Scale bar, 200 μm. (O and P) Altered OXT dynamics in CA2 and PrL across 5 consecutive days of chronic sleep disruption. In control animals, OXT signals in the CA2 during exploration of novel mice (O) and in the PrL during familiar mouse encounters (P) initially increased. In contrast, these responses progressively declined over time in SD-exposed animals. Notably, OXT fluorescence levels were importantly reduced in sleep-deprived mice compared to controls during novel mouse interactions in CA2 and familiar mouse interactions in PrL. Unpaired *t* tests were used for group comparisons (*n* = 8 mice per group). Box plots display medians, with whiskers indicating minimum and maximum values. Data are shown as means ± SEM; **P < 0.01 and ***P < 0.001.

We evaluated social recognition performance in chronically sleep-deprived mice using a 2-choice social interaction paradigm (Fig. 1A). In trial 1 (habituation), the experimental mouse was exposed to 2 empty cages for 5 min. In trial 2 (social memory encoding), 2 unfamiliar conspecifics (S1 and S2) were placed in the cages, and the mouse was allowed to freely investigate both for 5 min. After a 30-min delay, the mouse was returned to the arena for trial 3 (retrieval), during which one previously encountered mouse (S1 or S2) was replaced with a novel mouse (N), while the remaining familiar mouse (F) served as the familiar stimulus (S) (Fig. 1A): Discrimination index N = (time exploring mouse N − time exploring mouse S) / (time exploring mouse N + time exploring mouse S); Discrimination index F = (time exploring mouse S1 or S2 in in trial 2 − time exploring mouse S in trial 3) / (time exploring mouse S1 or S2 in in trial 2 + time exploring mouse S in trial 3). Under normal conditions, mice display intact social memory by (a) spending more time investigating the novel stimulus mouse (N) than the familiar one in trial 3 (reflected by a positive N index) and (b) reducing exploration time of the familiar mouse (S1 or S2) in trial 3 compared to trial 2 (captured by a positive F index).

In trial 3, sleep-deprived mice exhibited a marked reduction in exploration time toward the novel mouse (N) compared with controls, reflected by a lower discrimination index N, which persisted at least 24 d after SD induction (Fig. 1B). In addition, sleep-deprived mice showed more prolonged exploration of the familiar mouse, indicated by a reduced discrimination index F, which also persisted at least 24 d after SD induction (Fig. 1C). sleep-deprived mice did not show impairments in social exploration or novelty preference (Fig. S1). In acute sleep-deprived mice, social memory deficits were also observed but showed spontaneous recovery after 24 h (Fig. S2). These results suggest that chronic SD induces long-lasting social memory deficits.

### Chronic SD impaired OXT release in CA2 and PrL during social exploration

To investigate how chronic SD affects OXT signaling during social interactions, we used fiber photometry with a genetically encoded OXT sensor (GRAB_OXT1.8_; Fig. 1D to F) to monitor real-time OXT dynamics in the CA2 and the PrL. This sensor reports OXT binding via fluorescence changes resulting from conformational shifts in a G-protein-coupled-receptor-based scaffold, enabling direct observation of neuropeptide release [18]. In control mice, OXT release in the CA2 region was substantially elevated upon initial exposure to a novel conspecific but remained unchanged when exploring the familiar mice (Fig. 1G to J). In contrast, OXT release in the PrL was selectively increased during interactions with a familiar conspecific but not with a novel one (Fig. 1K to N). To rule out potential motion artifacts or nonspecific fluorescence changes unrelated to OXT binding, we conducted control experiments using an OXT-mutant sensor virus (OXTmut). This mutant sensor, which does not exhibit fluorescence changes upon OXT binding, was expressed in the CA2 and PrL regions under identical experimental conditions. No marked fluorescence fluctuations were detected in these control experiments (Fig. 1D to N), confirming that the observed signals in the experimental group were specifically attributable to OXT dynamics rather than behavioral motion or artifacts.

Under conditions of chronic SD, both brain regions exhibited an initial increase in OXT release upon social exposure, followed by a marked attenuation over time. Ultimately, OXT release in both the CA2 and PrL was reduced in sleep-deprived mice compared to controls (Fig. 1O and P). These findings suggest that OXT release in CA2 and PrL differentially encodes social novelty and familiarity, respectively. Both processes are significantly impaired under chronic SD conditions.

### Disruption of PVN^OXT^ neurons impaired social memory

**Fig. 2.**
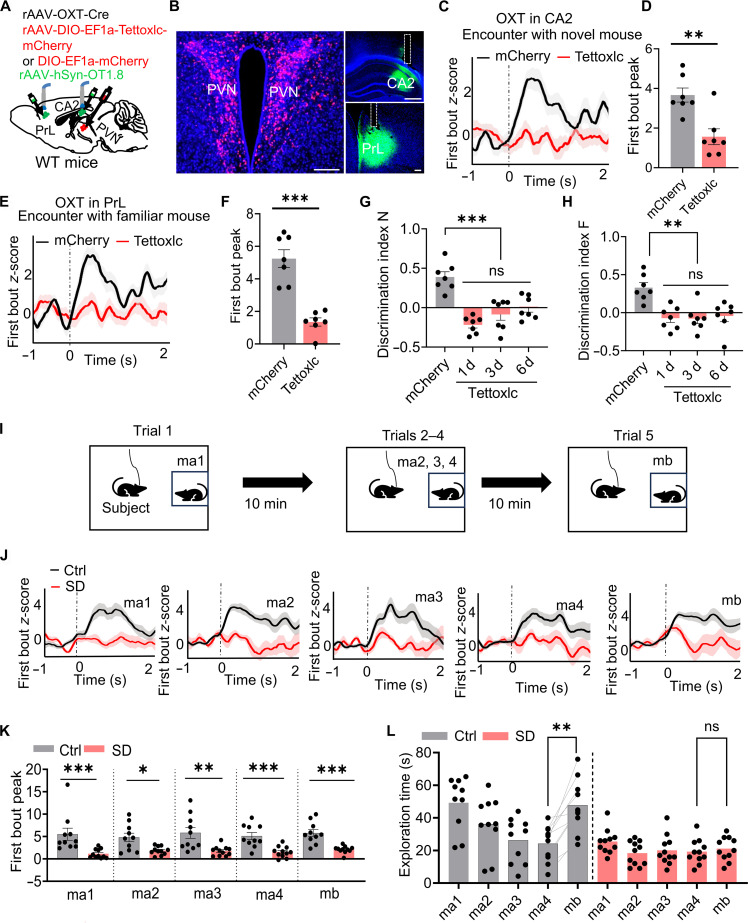
Dysfunction of PVN^OXT^ neurons resulted in social memory deficits. (A and B) Schematic showing Tettoxlc (red) expression in the bilateral PVN via rAAV-OXT-Cre and rAAV-DIO-EF1a-Tettoxlc-mCherry, alongside unilateral GRAB_OXT1.8_ biosensor expression in the CA2 or PrL via rAAV-hSyn-OT1.8 (green). Scale bars, 200 μm for PVN and PrL and 500 μm for CA2. (C to F) OXT traces during the first interaction bouts with novel (C and D) and familiar (E and F) mice aligned to the bout onset (time of 0 s) in the 2-choice social memory test. Reduced OXT signals in the CA2 and PrL were observed in the Tettoxlc group compared to mCherry group. Perievent plots display averaged fluorescence, with curves and shaded regions indicating the means ± SEM. Unpaired *t* test; *n* = 7 mice per group. (G and H) Tettoxlc mice showed reduced exploration of novel mice, indicated by a lower discrimination index N, and increased exploration of familiar mice, reflected in a lower discrimination index F, across days 1, 3, and 6. Two-way ANOVA analysis; *n* = 7 mice per group. (I) A progressive reduction in investigation of the repeated stimulus (ma1 to ma4) was observed, consistent with habituation, followed by a marked increase in exploration upon presentation of the novel mouse (mb). (J and K) The rAAV-OXT-Cre and rAAV-EF1α-DIO-GCaMP6m viruses were injected into bilateral PVN. *z*-scored GCaMP signals represent the averaged activity across all subject mice, time-locked to the onset of behavioral bouts (0 s). Sleep-deprived mice showed reduced Ca^2+^ activity during interactions with both familiar and novel mice compared to Ctrl. Perievent plots display averaged fluorescence, with curves and shaded regions indicating the means ± SEM. Unpaired *t* test; *n* = 10 mice in Ctrl group and *n* = 11 mice in SD group. (L) Sleep-deprived mice showed reduced habituation to the familiar mouse and impaired novelty recognition. Two-way ANOVA analysis; *n* = 10 mice in Ctrl group and *n* = 11 mice in SD group. Data are presented as means ± SEM, *P < 0.05, **P < 0.01, and ***P < 0.001. ns, no significant.

The PVN is a major source of OXT in the brain [23], with PVN^OXT^ neurons forming direct projections to both the CA2 and the PrL [24,25]. To determine whether OXT release from the PVN required for social memory, we utilized tetanus neurotoxin (Tettoxlc), a proteolytic agent that blocks synaptic vesicle fusion by cleaving vesicle-associated membrane proteins [26]. Recombinant adeno-associated virus (rAAV)-OXT-Cre was coinjected with a Cre-dependent Tettoxlc or mCherry virus (rAAV2/5-EF1a-DIO-Tettoxlc-mCherry or rAAV-EF1a-DIO-mCherry) bilaterally into the PVN. To monitor OXT signaling, we implanted the optical fibers above the CA2 or PrL and recorded the OXT dynamics using the GRAB_OXT1.8_ biosensor during a 2-choice social memory task (Fig. 2A and B). Mice expressing Tettoxlc exhibited a complete absence of novelty-induced OXT release in the CA2 and familiarity-induced release in the PrL in response to novel or familiar mice (Fig. 2C to F). Behaviorally, Tettoxlc-expressing mice also spent less time exploring the novel conspecific (N) compared to the familiar one (S1 or S2), resulting in a lower discrimination index N (Fig. 2G). In addition, during trial 3, these animals displayed increased investigation of the familiar mouse, a pattern that further lowered the discrimination index F (Fig. 2H). These impairments persisted over a 6-d follow-up period (Fig. 2G and H), indicating long-lasting impairments in social memory encoding and retrieval.

To further investigate the contribution of PVN^OXT^ neuronal activity to the social memory impairments observed after SD, we utilized a social-habituation-based paradigm. This protocol consisted of 4 repeated presentations of the same novel conspecific across successive trials (ma1 to ma4), followed by the introduction of a distinct unfamiliar mouse in the final trial (Fig. 2I). Under normal conditions, mice gradually reduce their investigation time toward the repeatedly encountered individual (habituation) and display renewed investigative behavior toward the newly introduced conspecific. The rAAV-OXT-Cre virus and Cre-inducible GCaMP6m virus (rAAV2/9-DIO-GCaMp6m) were bilaterally injected into the PVN. The control mice exhibited robust habituation to the repeated mouse and increased exploration of the novel one. In contrast, sleep-deprived mice showed important attenuated Ca^2+^ signals in PVN^OXT^ neurons during both novel and familiar interactions (Fig. 2J and K), accompanied by impaired social habituation, as indicated by reduced exploration of the novel mouse (Fig. 2L). These findings suggest that dysfunction of PVN^OXT^ neurons contributes to social memory deficits induced by SD.

### PVN^OXT^–CA2 and PVN^OXT^–PrL pathways respectively regulated social memory encoding and retrieval

**Fig. 3.**
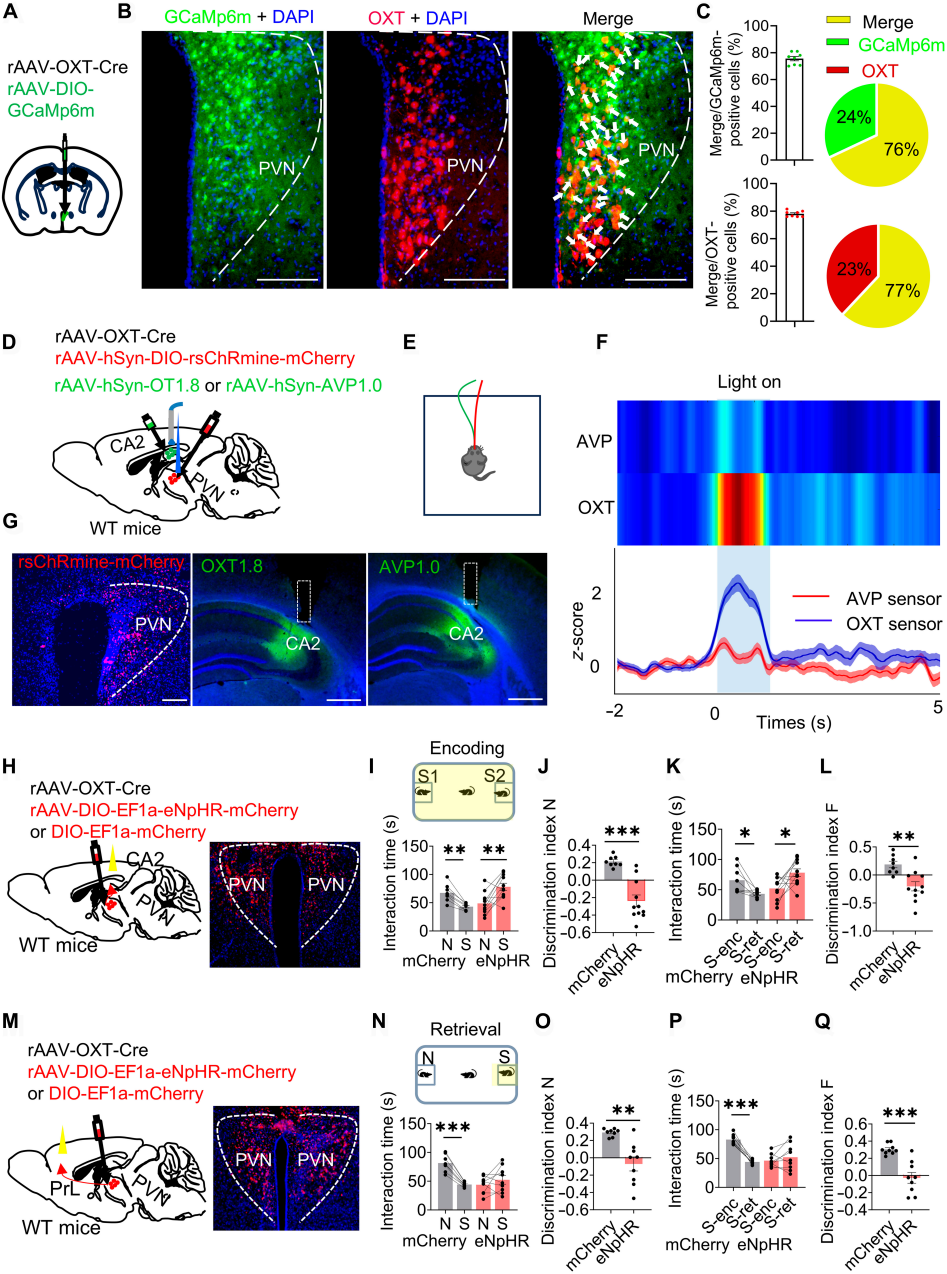
PVN^OXT^–CA2 and PVN^OXT^–PrL pathway respectively regulated social memory encoding and retrieval. (A) Diagram of unilateral viral infection using a mixture of rAAV-OXT-Cre and AAV-EF1α-DIO-GCaMP6m in the PVN. (B) Visualization of PVN^OXT^ neuron targeting by viral constructs. Mice received PVN injections of rAAV-OXT-Cre and AAV-DIO-hSyn-GCaMp6m to achieve selective labeling of OXT-expressing neurons. GCaMP6m expression (green) was Cre dependent, while OXT immunoreactivity (red) marked OXT^+^ cells. Overlapping fluorescence (yellow) in merged images confirmed the specificity of labeling in PVN^OXT^ neurons. Scale bars, 200 μm. (C) Merge cells versus GCaMP6m positive cells (left) or versus OXT-positive cells (right). *n* = 4 mice. (D to G) Ipsilateral expression of rsChRmine-mCherry in PVN^OXT^ neurons and GRAB_OXT1.8_ or GRAB_AVP1.0_ biosensor in CA2. Compared to the GRAB_AVP1.0_ group (*n* = 102 trials), OXT signaling in CA2 in the GRAB_OXT1.8_ group (*n* = 111 trials) was significantly enhanced in response to optogenetic stimulation (635 nm, 10-ms pulses at 10 Hz, 1-s on/50-s off, 20 min total; blue bars). *n* = 3 mice per group. Scale bars, 200 μm in PVN and 500 μm in CA2. (H to Q) Diagram of eNpHR or mCherry virus injection in PVN^OXT^ neurons and fiber implantation above CA2 (H) or PrL (M). Scale bars in (H) and (M) are the same as in (G). (I to L) Inhibition of the PVN^OXT^→CA2 pathway during the encoding phase (trial 2; eNpHR, *n* = 11; mCherry, *n* = 8) and (N to Q) inhibition of the PVN^OXT^→PrL pathway during the retrieval phase (trial 3; eNpHR, *n* = 9; mCherry, *n* = 8), both impaired social memory performance in eNpHR-expressing mice. N, exploration time for the novel mouse during retrieval; S, exploration time for the familiar mouse (S1/S2) during retrieval; S-enc, familiar mouse (S1/S2) exploration during encoding; S-ret, familiar mouse (S) exploration during retrieval. Data are shown as means ± SEM. *P < 0.05, **P < 0.01, and ***P < 0.001. Scale bars, 200 μm.

To delineate the respective roles of PVN^OXT^ neurons in social memory, we performed fiber photometry to monitor real-time Ca^2+^ dynamics in PVN^OXT^ neurons during social exploration. AAVs encoding OXT-promoter-driven Cre recombinase and Cre-dependent GCaMP6m were delivered into the PVN of wild-type (WT) mice (Fig. 3A). Immunohistochemical analysis revealed that approximately 80% of GCaMP6m-positive cells also expressed endogenous OXT, confirming the robust targeting specificity of the viral construct (Fig. 3B and C). To confirm whether the viral strategy selectively targeted OXT neurons without infecting potential arginine vasopressin (AVP)-expressing neurons, we performed a separate validation experiment using the same viral constructs (Fig. 3D to G). Ipsilateral expression of rsChRmine-mCherry was induced in PVN^OXT^ neurons, while the GRAB_OXT1.8_ or GRAB_AVP1.0_ biosensor was expressed in the CA2. Upon optogenetic stimulation (635 nm, 10-ms pulses at 10 Hz, 1-s on/50-s off, 20 min total; indicated by blue bars), OXT signaling in the GRAB_OXT1.8_ group (*n* = 111 trials) showed a significant enhancement compared to the GRAB_AVP1.0_ group (*n* = 102 trials). These results indicate minimal off-target infection of AVP neurons, supporting the specificity of the OXT-promoter-driven viral approach.

The Ca^2+^ imaging demonstrated that PVN^OXT^ neuronal activity is essential for both encoding and retrieval of social memory (Fig. S3A to H). To functionally interrogate these phases, we optogenetically inhibited PVN^OXT^ neurons by coexpressing rAAV-OXT-Cre with either rAAV-EFla-DIO-eNpHR3.0-mCherry or rAAV-EFla-DIO-mCherry virus in the PVN (Fig. S3I and J). The suppression of activity during either the encoding or retrieval window disrupted discrimination performance, as evidenced by comparable exploration times for familiar and unfamiliar conspecifics during the test (Fig. S3K to R).

We next examined projection-specific Ca^2+^ activity in PVN^OXT^ axon terminals using a Cre-dependent axon-targeted jGCaMP7b sensor (rAAV2/9-CAG-DIO-axon-jGCaMP7b-WPRE-hGH polyA) expressed in the PVN. Optical fibers were implanted unilaterally above either the CA2 (Fig. S4A and B) or PrL (Fig. S4E and F). PVN^OXT^ axon terminals within the CA2 displayed elevated Ca^2+^ transients during encounters with novel individuals (Fig. S4C and D), whereas PrL-targeting terminals preferentially responded to familiar partners (Fig. S4G and H).

These findings suggest a functional dissociation between the PVN^OXT^–CA2 and PVN^OXT^–PrL projections, with the former primarily engaged in social memory formation and the latter supporting memory retrieval. To further distinguish the subpopulations of PVN^OXT^ neurons projecting to CA2 versus PrL, we used 2 independent viral tracing strategies. In the first approach (Fig. S5A to E), we unilaterally injected the retrograde tracer rAAV2/R-AAV-DIO-hSyn-EGFP into the PrL and rAAV2/R-AAV-DIO-hSyn-mCherry into the CA2 of the same hemisphere, along with an ipsilateral injection of AAV-OXT-Cre into the PVN (*n* = 5 mice). Histological analysis of coronal sections across 3 rostrocaudal levels revealed that OXT neurons projecting to CA2 (red) and those projecting to the PrL (green) showed minimal overlap, indicating largely segregated neuronal populations. In the second approach (Fig. S5F to I), we unilaterally injected the retrograde Cre-driver virus rAAV2/R-OXTR-Cre into either the CA2 (*n* = 2 mice) or the PrL (*n* = 2 mice), combined with bilateral injections of the Cre-dependent reporter AAV-DIO-hSyn-GCaMP6m into the PVN. Coronal sections were prepared from anterior to posterior relative to bregma for histological verification. Subtype classification confirmed that PVN^OXT^ neurons projecting to CA2 and PrL constitute distinct subpopulations. A subset of PVN^OXT^ neurons projects to the contralateral CA2 or PrL, and these projecting populations are largely nonoverlapping. Furthermore, we observed a limited number of neurons with collateral projections to both regions, suggesting the presence of divergent axonal branching within the PVN^OXT^ circuit (Fig. S5H and I).

To examine the role of the PVN^OXT^–CA2 pathway in social memory encoding, we bilaterally injected mice into the PVN with rAAV-OXT-Cre and either a Cre-inducible eNpHR-mCherry or mCherry control virus (rAAV-DIO-EF1a-eNpHR-mCherry or rAAV-DIO-EF1a-mCherry). Optical fibers were implanted unilaterally above the CA2 (Fig. 3H). Control animals expressing mCherry exhibited intact discrimination behavior, preferentially investigating the novel over the familiar conspecific during trial 3, accompanied by a decline in interaction time with the previously encountered mouse from trial 2. In contrast, photoinhibition of CA2 during the encoding phase in eNpHR-expressing mice disrupted this novelty preference, as evidenced by diminished investigation of the novel mouse and sustained engagement with the familiar individual (Fig. 3I to L). Notably, light delivery restricted to the retrieval phase did not alter behavior, supporting a selective role for the PVN^OXT^–CA2 projection in memory acquisition rather than recall (Fig. S6A to E).

We next investigated how disrupting PVN^OXT^–PrL communication during exposure to familiar conspecifics would influence memory retrieval. Mice were bilaterally injected in the PVN with rAAV-OXT-Cre along with either rAAV-DIO-EF1a-eNpHR3.0-mCherry or a control virus (rAAV-DIO-EF1a-mCherry), and fiber-optic cannulas were placed above the PrL (Fig. 3M). Relative to controls, mice expressing eNpHR3.0 failed to distinguish between novel and familiar conspecifics during the retrieval test, consistent with impaired access to stored social representations. These animals also maintained elevated interaction levels with the familiar stimulus from the encoding phase (Fig. 3N to Q), reinforcing the retrieval-specific nature of this deficit. Importantly, inhibition of the same pathway during the encoding phase had no observable effect on memory formation (Fig. S6G and H). Surprisingly, optogenetic activation of PVN^OXT^ projections to the PrL during encoding similarly disrupted memory performance (Fig. S6I and J), underscoring the importance of temporally precise regulation of this pathway for appropriate memory retrieval.

### Optogenetic activation of PVN^OXT^–CA2 and PVN^OXT^–PrL transiently improved social memory deficits in chronic sleep-deprived mice

**Fig. 4.**
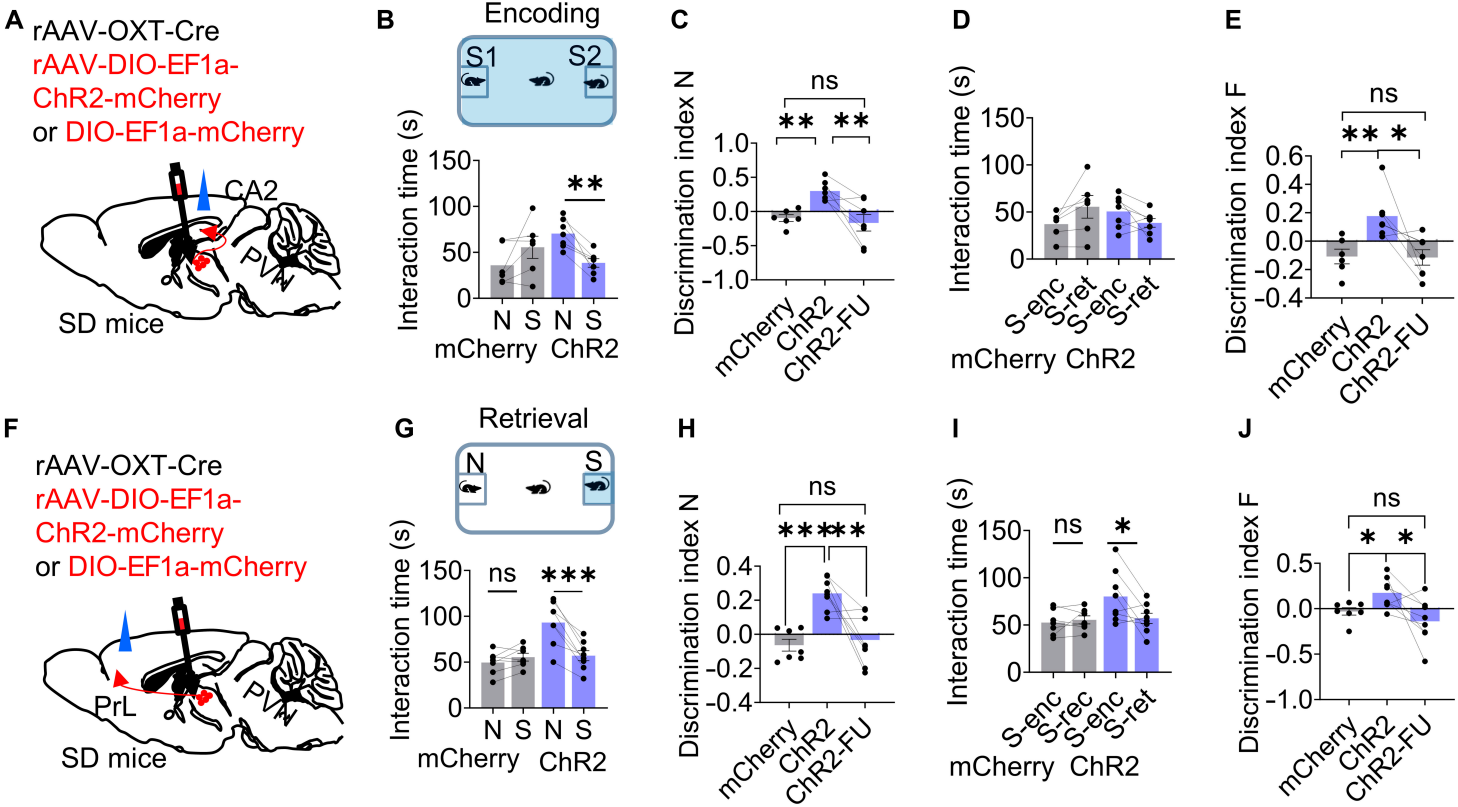
Optogenetic activation of PVN^OXT^–CA2 and PVN^OXT^–PrL transiently improved social memory deficits in sleep-deprived mice. (A to J) Diagram of ChR2 or mCherry virus injection in PVN^OXT^ neurons and fiber implantation above CA2 (A) or PrL (F). Optogenetic activation of PVN^OXT^–CA2 pathway during the encoding phase (trial 2; ChR2, *n* = 7 mice; mCherry, *n* = 6 mice) (B to E) or photoactivation of PVN^OXT^–PrL pathway during the retrieval phase (trial 3; ChR2, *n* = 8 mice; mCherry, *n* = 7 mice) (G to J) in 2-choice social memory test, improved social memory performance of animals expressing ChR2 in PVN^OXT^ relative to the control group expressing mCherry in SD mice. ChR2-FU, social memory of mice in the ChR2 group was followed up after 24 h. Data are presented as means ± SEM. *P < 0.05, **P < 0.01, and ***P < 0.001.

Given the robust activation of PVN^OXT^–CA2 projections during interactions with novel mice, we investigated whether optogenetic activation of this pathway could ameliorate social memory deficits in chronically sleep-deprived mice. Mice were injected bilaterally in the PVN with rAAV-OXT-Cre along with either a Cre-dependent ChR2-mCherry or control mCherry virus (rAAV2/9-DIO-EF1a-ChR2-mCherry or rAAV2/9-DIO-EF1a-mCherry), and optical fibers were implanted unilaterally above the CA2. Control sleep-deprived mice (mCherry group) failed to discriminate between novel and familiar individuals in trial 3 (Fig. 4B and C) and also maintained elevated investigation time toward the familiar mouse previously encountered in trial 2 (Fig. 4D and E). Optogenetic stimulation of the PVN^OXT^–CA2 projection during the encoding phase marked restored memory performance in ChR2-expressing animals, which displayed enhanced novelty preference in trial 3 (Fig. 4B and C) and spent markedly less time with the familiar conspecific (Fig. 4D and E).

However, this improvement was transient, as performance declined and deficits reappeared within 24 h poststimulation (Fig. 4C and E). These results indicate that temporally targeted activation of PVN^OXT^–CA2 projections during encoding can transiently counteract SD-induced disruptions in social memory. Although anatomical evidence indicates that OXT neurons in the PVN also project to the hippocampal CA3 region, this pathway appears not required for social memory regulation. Notably, simultaneous inhibition of CA3 function during activation of the PVN^OXT^–CA2 pathway still rescued social memory deficits in sleep-deprived mice (Fig. S7A to D). These results suggest that PVN^OXT^ neurons primarily regulate social memory through CA2 rather than CA3, indicating clear subregional specificity of this circuit in the encoding of social memory.

Since PVN-PrL projections were robustly activated during encounters with familiar mice, we next investigated the effects of activating the PVN^OXT^–PrL pathway during interactions with familiar mice. Bilateral PVN delivery of rAAV-OXT-Cre along with either rAAV-DIO-EF1a-ChR2-mCherry or the control mCherry vector was performed, and fibers were positioned above the PrL (Fig. 4F). Compared to control mice, ChR2 mice demonstrated improved social memory, showing greater preference for the novel conspecific in trial 3 (Fig. 4G and H) and reduced familiarity-driven engagement (Fig. 4I and J). As observed with CA2-targeted activation, the enhancement was short-lived, with behavioral deficits resurfacing by 24 h after light stimulation. Together, these findings suggest that while activation of PVN^OXT^–CA2 and PVN^OXT^–PrL circuits can temporarily restore social memory function in sleep-deprived mice, the underlying impairment remains unresolved and resurfaces over time.

### High-frequency optogenetic activation of PVN^OXT^ neurons contributed to sustained improvement in social memory

Considering that chronic SD impair PVN^OXT^ neuron activity, effective strategies to restore this activity are crucial for mitigating social memory deficits. A prior study suggested that inducing short-term hyperpolarization could transiently enhance neuronal function [27]. Following this approach, we injected AAVs (rAAV-OXT-Cre) and either Cre-inducible eNpHR or mCherry control virus (rAAV-DIO-EF1a-eNpHR-mCherry or rAAV-DIO-EF1a-mCherry) bilaterally into the PVN, implanting optical fibers above the PVN. However, using a protocol of described previously (7 s × 10 with a 25-s interstimulus interval daily for 3 consecutive days), we observed no marked improvement in social memory was observed in sleep-deprived mice (Fig. S8A to E).

**Fig. 5.**
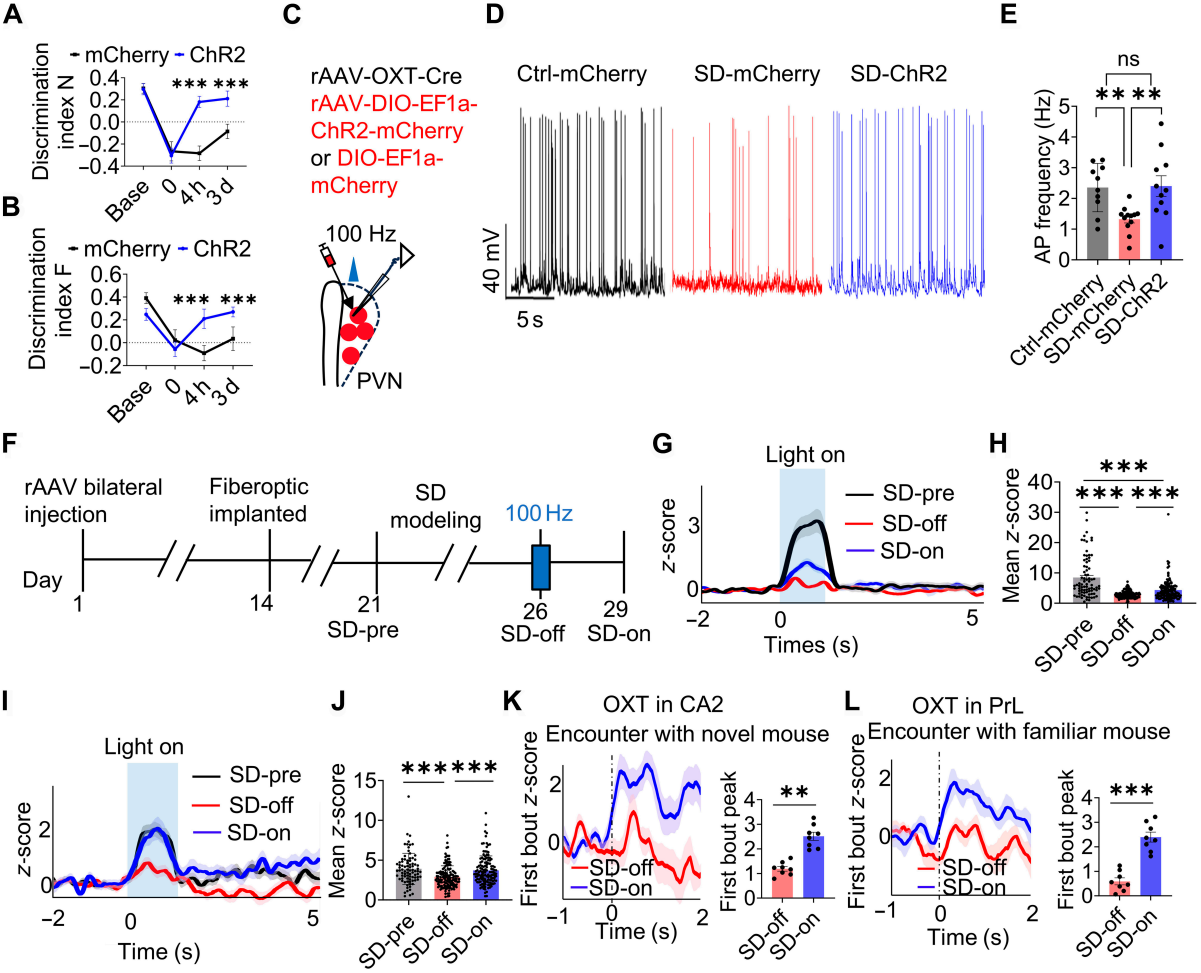
High-frequency optogenetic activation of PVN^OXT^ neurons contributed to sustained improvement in social memory deficits. (A and B) The social memory of all subject mice was investigated using the “two-choice social memory test”. ChR2 group mice spent more time exploring the novel mice N with a continued higher discriminant index N after 4 h and 3 d (A). ChR2 group mice spent less time exploring familiar mouse S with a continued higher discriminant index F after 4 h and 3 d (B). Base, 1 d before sleep disruption begins; 0, at 7:00 PM on the final day of chronic SD. Two-way ANOVA analysis; *n* = 11 mice in mCherry group and *n* = 18 mice in ChR2 group. Box plots show the median and whiskers representing the minimum and maximum values. (C to E) Increased action potential (AP) frequency in PVN^OXT^ neurons was observed 3 d after 100-Hz stimulation in sleep-deprived mice. (C) Schematic showing whole-cell recordings from PVN^OXT^ neurons transduced with a combination of rAAV-OXT-Cre and either rAAV-DIO-EF1a-ChR2-mCherry or rAAV-DIO-EF1a-mCherry using a patch pipette. (D) Representative voltage traces recorded in Ctrl-mCherry, SD-mCherry, and SD-ChR2 groups. (E) Chronic sleep disruption reduced the spontaneous firing rate of PVN^OXT^ neurons in the SD-mCherry group, whereas a higher firing rate was restored in the SD-ChR2 group after 100-Hz stimulation, showing no marked difference from the Ctrl-mCherry group. Recordings were performed 3 d after stimulation. Ctrl-mCherry, *n* = 10 cells; SD-mCherry, *n* = 12 cells; SD-ChR2, *n* = 11 cells. One-way ANOVA; *n* = 3 mice per group. (F) Experimental timeline in (G) to (L). We applied the 100-Hz treatment after completing the tests in the SD-off group and allowing the mice to rest for 4 h. SD-pre, before SD; SD-off, 4 h before the 100-Hz stimulus in sleep-deprived mice.; SD-on, day 3 after 100-Hz intervention in sleep-deprived mice. (G and H) Bilateral expression of ChR2-mCherry in PVN^OXT^ neurons and unilateral expression of GRAB_OXT1.8_ biosensor in CA2. Compared to the SD-off group (*n* = 132 trials), OXT signaling in CA2 in the SD-pre (*n* = 80 trials) and SD-on (*n* = 131 trials) groups was significantly enhanced in response to optogenetic stimulation (473 nm, 10-ms pulses at 10 Hz, 1-s on/50-s off, 20 min total; blue bars). Significant difference was observed between SD-pre and SD-on. One-way ANOVA; Ctrl, *n* = 3 mice; other groups, *n* = 4 mice. (I and J) Bilateral ChR2-mCherry expression in PVN^OXT^ neurons and unilateral GRAB_OXT1.8_ expression in PrL. Compared to the SD-off group (*n* = 136 trials), the OXT signals in the PrL of SD-pre (*n* = 84 trials) and SD-on (*n* = 132 trials) mice were also elevated, but no significant difference was found between SD-pre and SD-on groups. One-way ANOVA; *n* = 4 mice per group. (K and L) Compared to SD-off group, higher peak *z*-score of first interaction of OXT release in CA2 (K) and PrL (L) was observed in SD-on group during social exploration. Paired *t* test; *n* = 8 mice per group. Data are presented as means ± SEM. **P < 0.01 and ***P < 0.001.

Given the lack of effect with inhibitory stimulation, we turned to an excitatory strategy. High-frequency stimulation of specific neural circuits has previously been shown to restore neuronal excitability and reverse behavioral deficits in both preclinical and clinical studies [28,29]. Building on this, we applied high-frequency optogenetic stimulation (100 Hz) in sleep-deprived mice using a previously established protocol (~5 mW, 473 nm), which consisted of 5 trains of 100-Hz light pulses (1 s per train, 2-ms pulse width), delivered at 15-s intervals. To enable pathway-specific manipulation, we coinjected rAAV-OXT-Cre with either a Cre-dependent ChR2-mCherry or a control mCherry virus (rAAV-DIO-EF1a-ChR2-mCherry or rAAV-DIO-EF1a-mCherry) bilaterally into the PVN. Notably, behavioral assessments conducted 3 d after stimulation revealed robust and persistent restoration of social memory in ChR2-expressing mice (Fig. 5A and B), a benefit consistently reproduced across 2 additional behavioral paradigms (Fig. S9A to E).

To investigate the cellular mechanisms underlying this effect, we conducted whole-cell electrophysiological recordings in PVN^OXT^ neurons. Chronic SD markedly suppressed spontaneous firing, but this was rescued by 100-Hz photostimulation in ChR2-expressing mice, restoring firing rates to levels comparable to nondeprived controls (Fig. 5C to E). Thus, high-frequency stimulation effectively rescues the hypoactivity of PVN^OXT^ neurons induced by SD, providing a plausible cellular basis for the sustained behavioral recovery.

Furthermore, the transmission in PVN^OXT^–CA2 and PVN^OXT^–PrL neural pathways were investigated. For this, rAAV-OXT-Cre and rAAV-DIO-ChR2-mCherry were delivered into the PVN, while the GRAB_OXT1.8_ sensor was unilaterally expressed in either the CA2 or PrL. We found that the OXT signals in CA2 (Fig. 5G to H) or PrL (Fig. 5I and J) were more active after the optogenetic stimulation of PVN^OXT^ neurons (SD-pre versus SD-off and SD-off versus SD-on). In addition, to detect OXT signals in CA2 and PrL of sleep-deprived mice during social interaction before and after 100-Hz treatment of sleep-deprived mice, the GRAB_OXT1.8_ biosensor virus (rAAV-hSyn-OT1.8) was injected into CA2 or PrL. Compared to SD-off group, a higher OXT fluorescence signal was investigated in the CA2 (Fig. 5K) and PrL (Fig. 5L) in SD-on group during social exploration. Together, these results suggested that 100-Hz photostimulation of PVN^OXT^ neurons could consistently improve social memory deficits of sleep-deprived mice by enhancing PVN^OXT^ neuronal activity and increasing OXT release in the CA2 and PrL.

## Discussion

**Fig. 6.**
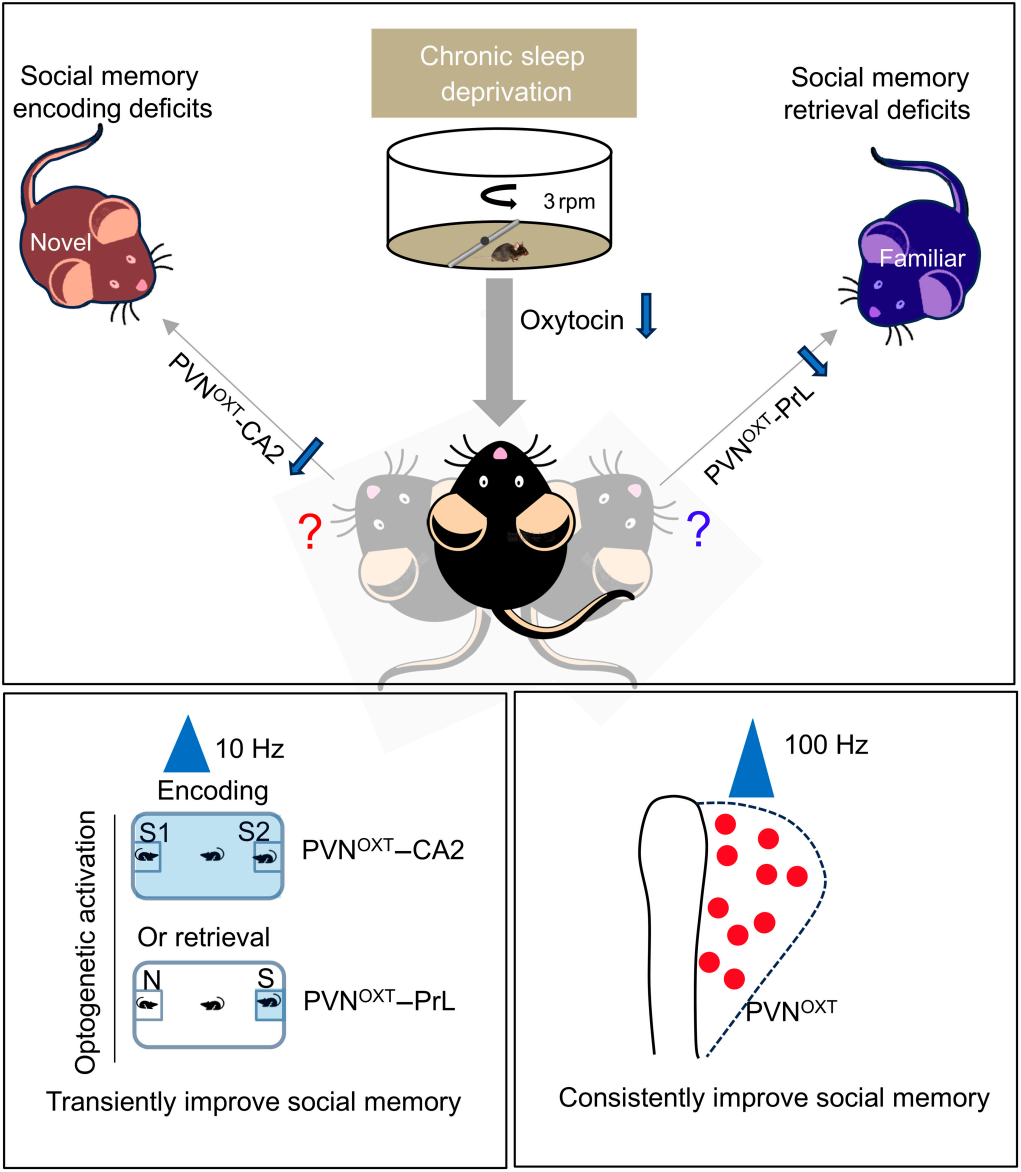
Working model. Chronic sleep disruption impairs the function of both the PVN^OXT^–CA2 and PVN^OXT^–PrL neural circuits and their OXT release, leading to defects in social memory encoding and retrieval. By optogenetically activating the PVN^OXT^–CA2 and PVN^OXT^–PrL neural circuits at 10 Hz during the encoding and retrieval phases of social memory, temporarily social memory improvement in sleep-deprived mice was achieved. However, high-frequency (100-Hz) optogenetic activation of PVN^OXT^ neurons leads to sustained improvements in social memory.

Social memory deficits associated with sleep disorders are frequently observed in various neurological conditions, yet the underlying neurocircuit and molecular mechanisms remain poorly understood. In our study, we found that PVN^OXT^–CA2 and PVN^OXT^–PrL pathways distinctly regulate the encoding and retrieval phases of social memory, respectively, each exhibiting different OXT response patterns. We demonstrate that SD induces decrease in PVN^OXT^ neuronal activity, thereby impairing PVN^OXT^–CA2, PVN^OXT^–PrL circuit and leading to reduced OXT release (Fig. 6). This impairment can be transiently ameliorated by pathway-specific, time-locked optogenetic activation, stimulating the PVN^OXT^–CA2 pathway during encoding or the PVN^OXT^–PrL pathway during retrieval. In contrast, high-frequency optogenetic activation of PVN^OXT^ neurons sustainably rescued SD-induced social memory deficits. Overall, our findings provide new insights into the interplay between neuropeptide signaling and neural circuitry in social memory and suggest potential therapeutic strategies for treating social memory deficits associated with sleep disruption.

Social memory deficits are common in patients with ASD [1], AD [2], and PTSD [30–32], conditions often accompanied by sleep disturbances [3–5]. OXT administration has been shown to enhance social competence in patients with ASD [33] and holds therapeutic potential for PTSD [34] and AD [2]. By leveraging the high-resolution GRAB_OXT1.8_ sensor, we mapped the precise spatiotemporal dynamics of OXT release during distinct stages of social memory processing—encoding and retrieval—mediated through the CA2 and PrL circuits, respectively. Furthermore, we underscore the therapeutic promise of targeting OXT signaling, potentially via activity-enhancing neuromodulation, to treat social cognitive impairments across a range of neuropsychiatric conditions associated with SD.

In the hippocampus, evidence indicates that OXT primarily facilitates social recognition via OXTR, whereas arginine vasopressin (AVP) modulates stress- and aggression-related behaviors through AVPR1a and AVPR1b receptors [35]. The GRAB_OXT1.0_ sensor demonstrates approximately 12-fold higher affinity for OXT than for AVP in cultured cells [18]. Modifications to the ligand-binding pocket of the GRAB_OXT1.8_ sensor have markedly enhanced its affinity for OXT, exceeding that of GRAB_OXT1.0_, while improving specificity and binding efficacy. Despite its optimization for higher affinity and specificity in OXT binding compared to GRAB_OT1.0_, GRAB_OXT1.8_ may still exhibit some cross-reactivity with AVP. This limitation should be considered when interpreting OXT release data in regions such as CA2 and PrL, as AVP signaling might also influence the observed effects [10]. Moving forward, further investigation into the role of AVP in social memory deficits following SD is warranted. Future studies should use more selective monitoring and manipulation tools to dissect the distinct and potentially overlapping contributions of OXT and AVP signaling.

During chronic SD, an initial transient increase in OXT release was observed in the CA2 and PrL regions, followed by a marked decline over time. This pattern likely represents an early compensatory response to SD that diminishes with prolonged SD. Figure 2 shows the reduction in OXT release during SD, which correlates with social memory deficits but does not establish causation. In contrast, Fig. 4 presents direct evidence that restoring OXT release alleviates these impairments, supporting the hypothesis that SD-induced reductions in OXT release underlie social memory deficits. However, reduced OXT release might also be a downstream consequence of these deficits rather than their primary cause, which should be considered. Further investigation is warranted to determine whether diminished OXT release serves as a driving factor or a downstream correlate of SD-induced social memory impairment. Using causal manipulations with temporally precise control of OXT signaling could help disentangle these possibilities.

Within the hippocampal CA2 region, PYR neurons play a critical role in encoding social novelty [7,8]. During the exploration of novel conspecifics, the activity of vasoactive intestinal peptide (VIP) interneurons in the CA2 region increases selectively, inducing long-term depression of feedforward inhibition onto CA2 PYR neurons [36]. Activation of OXTRs enhances the responsiveness of CA2 PYR neurons by refining their synaptic inputs [17]. OXT can also modulates downstream neuronal activity through diverse mechanisms [37], potentially regulating CA2-mediated social memory encoding either directly through PYR neurons or indirectly through VIP neurons. However, the precise sequence of events and pathways involved remains to be elucidated. In addition, PVN^OXT^ axons were identified in the dorsal CA3 region. Although deletion of OXTRs in CA3 impairs social recognition memory [16], chemogenetic silencing of dorsal CA3 does not importantly affect social memory [38]. Remarkably, activation of the PVN^OXT^–CA2 pathway during social memory encoding improved social memory even when CA3 was chemically inhibited. These findings suggest that the PVN^OXT^–CA2 pathway operates independently of CA3 in social memory encoding, warranting further investigation into the underlying mechanisms.

Activation of the PVN^OXT^–PrL pathway in our study enabled mice to recall “forgotten” social memories, distinguishing novel conspecifics from familiar ones during the retrieval phase. In contrast, activating this pathway during the encoding phase impaired social memory, aligning with findings that 3 consecutive days of restraint stress activate PVN^OXT^ neurons and disrupt social memory in mice [39]. These results suggest that abnormally elevated OXT concentrations during memory encoding, driven by overactivation of the PVN^OXT^–PrL pathway, may lead to social memory deficits. In addition, prior work has shown that optogenetic suppression of cholecystokinin (CCK)-positive interneurons in the PFC disrupts working memory retrieval [40]. Our preliminary findings indicate a potential role for PrL CCK interneurons in mediating OXT-dependent social memory recall (Fig. S10A to L), shedding light on a putative cellular mechanism by which the PVN^OXT^–PrL circuit influences memory retrieval. Our retrograde tracing data (Fig. S5) reveal that PVN^OXT^ neurons projecting to CA2 and PrL are largely distinct populations, with an additional subset exhibiting bilateral collateral projections—particularly to CA2. While most neurons are target-specific, these anatomical differences suggest a structural basis for their differential functional roles: The PVN^OXT^–CA2 pathway appears primarily involved in social memory encoding, whereas the PVN^OXT^–PrL pathway is more engaged in retrieval, likely due to distinct circuit mechanisms and OXT release patterns in each region.

Although mimicking short-term hyperpolarization in neurons induces sustained activation and antidepressant-like effects in animal models [27], this approach failed to reverse memory deficits in sleep-deprived mice. This likely reflects the unique electrophysiological properties of PVN^OXT^ neurons, which seem resistant to short-term hyperpolarization. A balanced stimulation protocol targeting both encoding and retrieval phases may be required to fully restore social memory in sleep-deprived mice.

Noninvasive neurostimulation techniques hold promise for mitigating cognitive decline [28,41]. For example, high-frequency electrical stimulation of the posterior insula acutely raises pain thresholds [42]. Similarly, our data show that 100-Hz optogenetic activation of PVN^OXT^ neurons markedly improves social memory in sleep-deprived mice, potentially through 3 distinct mechanisms: (a) 100-Hz stimulation may synchronize neuronal populations, enhancing information transfer by aligning firing patterns; (b) it may regulate ion channel activity, affecting sodium, potassium, or calcium flux that is critical for action potential propagation; and (c) it may induce short-term synaptic plasticity, increasing neurotransmitter release or postsynaptic responses to enhance neuronal firing rates. Clinical studies indicate that neurostimulation can alleviate pathological symptoms, although its benefits often diminish or worsen after cessation [43]. This highlights the need to further optimize neurostimulation parameters and precisely target brain regions for therapeutic interventions [44]. Future research should examine the mechanisms of 100-Hz optogenetic stimulation and focus on developing noninvasive techniques. In contrast, low-frequency stimulation at 4 Hz does not produce these effects (Fig. S8F to H). Our findings demonstrate that optogenetic stimulation of PVN^OXT^ neurons restores social memory deficits induced by sleep-deprived. Activation of PVN^OXT^ neurons triggers OXT release via the 2 circuits studied, but the possibility of broader, global OXT release should not be overlooked. Given the extensive projections of OXT neurons to multiple brain regions [37], global OXT release, rather than localized circuit-specific effects, likely contributes to the observed recovery. Although our investigation centers on the CA2 and PrL pathways, future work should explore the differential contributions of localized circuit-level versus widespread OXT signaling to the observed memory outcomes.

Our results are further corroborated by a recent study by Liu et al. [45], which utilized magnetic-nanoparticle-mediated stimulation to specifically activate PVN^OXT^ neurons and observed marked improvement in social behavior in a mouse model of ASD. This supports the notion that targeted enhancement of PVN^OXT^ neuronal activity may offer a therapeutic avenue for mitigating social memory deficits. Notably, the noninvasive methodology used by Liu et al [45]. suggests a potentially viable translational route beyond conventional optogenetic approaches, which are currently limited to experimental settings. We fully acknowledge that the therapeutic application of precise OXT neuron modulation remains at an early stage. Several key challenges must be addressed before clinical translation can be considered, including a more comprehensive understanding of the broader neurochemical impacts and network-level adaptations resulting from such interventions. For instance, the influence of OXT neuron activation on correlated neurotransmitter systems and the long-term stability of induced circuit changes remain largely unexplored. Thus, we should consider these mechanistic and translational limitations and highlight the necessity of future studies investigating stimulation parameters—such as frequency and temporal pattern—which may critically influence treatment efficacy and network-level outcomes.

In our study, chronic sleep-deprived mice exhibited a negative discrimination index in the 2-choice social memory test, indicating importantly longer exploration time toward familiar conspecifics compared to novel ones. Although this result is consistent with previous reports [46], its behavioral implications may extend beyond a mere loss of social memory. The lack of a clear preference around zero discrimination index suggests the possible involvement of more complex cognitive and/or affective mechanisms: (a) altered reconstitution of social memory representations, (b) impaired detection or evaluation of novel stimuli, (c) a motivation–affection shift leading to excessive preference for familiar stimuli. Our previous work has also shown that chronic SD can induce pronounced anxiety-like behaviors in mice [47]. Thus, we propose that such an anxiety-like state may drive sleep-deprived mice to favor safer and more familiar social stimuli, thereby leading to aberrant social preference patterns. These findings provide new insights into how chronic SD may affect social cognition, although the underlying neural circuits and molecular mechanisms require further investigation.

Several limitations of the present study should be noted. First, the viral construct driven by the OXT promoter achieved approximately 76% to 80% specificity in labeling OXT-expressing neurons, which is consistent with prior studies using similar approaches. Although this level of specificity is generally acceptable for functional studies, it does not completely exclude the possibility of labeling a minority of nonoxytocinergic neurons. Hence, we refer to these as “predominantly OXT-expressing neurons” throughout the manuscript. Future studies using OXT-Cre mouse lines could improve targeting precision. Second, the OXT antibody used in this study may cross-react with AVP. Given that vasopressinergic signaling is also implicated in social memory [10], future work should directly compare the roles of OXT and AVP to clarify the specificity of OXT-mediated effects and potential interactions between these pathways. Furthermore, the unilateral optogenetic approach may underestimate the full effect of bilateral OXT circuit modulation, highlighting the need for future studies using bilateral interventions to fully elucidate the mechanisms of social memory. In addition, the study was conducted exclusively in male mice to avoid confounding variables from the female estrous cycle, which modulates neuronal excitability, social behavior, and OXTR expression. We acknowledge this limits generalizability to females, and studying these circuits in females is a critical future direction. Finally, due to the large anatomical size of CA3 and limited viral spread, our inhibition of this region was incomplete. To more precisely dissect hippocampal subregion contributions, future studies using cell-type-specific OXTR knockouts alongside targeted PVN^OXT^–CA2 activation will be valuable.

## Conclusion

Our findings demonstrate that chronic SD disrupts OXT release in a circuit-specific manner, leading to persistent social memory deficits. We identified distinct roles for the PVN^OXT^–CA2 and PVN^OXT^–PrL pathways in encoding and retrieving social memory, respectively. While time-locked activation of these downstream pathways transiently rescued memory, high-frequency optogenetic stimulation (100 Hz) of PVN^OXT^ neurons themselves induced a sustained recovery, highlighting restoration of the neuronal source as a more effective strategy.

This study underscores the potential of targeted neuromodulation to treat circuit-specific deficits. Beyond clarifying the neuropeptidergic basis of sleep-related social memory impairment, our study has broader translational implications. Social memory dysfunction is an early feature of conditions such as AD. The integration of multimodal-biomarker-based predictive models, combining plasma analytes and magnetic resonance imaging radiomics, may improve the precision of cognitive decline assessment [48,49]. In this context, the development of highly sensitive biosensors [50] based on fluorescent probes holds great promise for the early detection and targeted intervention of social memory dysfunction, thereby facilitating earlier diagnosis and enabling personalized therapeutic strategies for AD. Thus, enhancing OXT signaling in specific brain circuits emerges as a novel therapeutic avenue for social cognitive deficits across multiple neuropsychiatric disorders associated with sleep disruption.

## Methods

### Animals

WT C57BL/6J mice (8 to 12 weeks, 22 to 28 g) and CCK-Cre mice (8 to 12 weeks, 23 to 26 g) were obtained from Beijing Vital River Laboratory Animal Technology Co. Ltd. Only male mice were used in this study because there were significant differences between male and female mice [51,52] in social competence and social cognition, which would have complicated the analyses in this study. All animals were housed in cages at an ambient temperature of 22 ± 0.5 °C and a relative humidity level of 60% ± 2% on an automatically controlled 12-h light/dark cycle, with lights on at zeitgeber time 0 (ZT 0; 7:00). Animals were maintained in individual cages under standard conditions with free access to food and water.

### Stereotaxic surgery

Under 1% pentobarbital sodium anesthesia (75 mg/kg, intraperitoneally), mice were secured in a stereotaxic apparatus (RWD, China). Viral vectors (50 nl) were injected at 17 nl/min via a glass micropipette using a precision microinjection pump (Pump11 Elite Nanomite, Harvard Apparatus). The pipette was retained in position for 8 min postinfusion before being carefully withdrawn.

To monitor OXT dynamics in the CA2 or PrL, rAAV9-hSyn-OT1.8 (titer, 3.08 × 10^12^ vg/ml; Brain Case Co. Ltd., Wuhan) was stereotaxically delivered into either the CA2 (anteroposterior [AP], −1.82 mm; mediolateral [ML], ±2.0 mm; dorsoventral [DV], −1.68 mm) or the PrL (AP, 1.95 mm; ML, ±0.30 mm; DV, −2.2 mm). rAAV9-hSyn-AVP1.0 was used in our work (titer, 2.08 × 10^12^ vg/ml; Brain Case Co. Ltd., Wuhan). To suppress OXT release from PVN, a viral cocktail consisting of rAAV2/5-EF1a-DIO-Tettoxlc-P2A-mCherry-WPREs (2.47 × 10^12^ vg/ml) and rAAV2/9-OXT-Cre-WPRE-hGH-pA (5.16 × 10^12^ vg/ml) was injected into the PVN (AP, −0.65 mm; ML, ±0.2 mm; DV, −4.80 mm; Brain VTA Co. Ltd., Wuhan). For optogenetic stimulation or inhibition and for fiber photometry analyses, the following Cre-dependent viral vectors were used: rAAV2/9-DIO-EF1a-hChR2(H134R)-mCherry-WPRE-pA (2.68 × 10^12^ vg/ml), rAAV2/9-DIO-EF1a-eNpHR-mCherry-WPRE-pA (1.17 × 10^12^ vg/ml), rAAV2/9-DIO-hSyn-GCaMP6m-WPRE-pA (5.54 × 10^12^ vg/ml), and CaMKIIa-hM4D(Gi)-mCherry-WPRE-hGHpA (5.38 × 10^12^ vg/ml) (all from Brain VTA Co. Ltd., Wuhan). To measure the axonal calcium activity of PVN^OXT^ neurons projecting to either CA2 or PrL, rAAV2/9-CAG-DIO-axon-jGCaMP7b (2.30 × 10^12^ vg/ml) was administered into the PVN.

To further identify subpopulations of PVN^OXT^ neurons, rAAV2/R-OXTR-CRE-WPRE-hGHpA (2.00 × 10^12^ vg/ml; Brain VTA Co. Ltd., Wuhan, China) was stereotaxically delivered into either the CA2 or PrL through 3 separate injections along the anteroposterior axis. For CA2 targeting, the coordinates (relative to bregma) were as follows: injection 1: AP, −1.70 mm; ML, ±2.0 mm; DV, −1.68 mm; injection 2: AP, −1.82 mm; ML, ±2.50 mm; DV, −1.80 mm; injection 3: AP, −2.30 mm; ML, ±2.70 mm; DV, −1.95 mm. For PrL targeting, the coordinates were as follows: injection 1: AP, +2.10 mm; ML, ±0.30 mm; DV, −2.00 mm; injection 2: AP, +1.95 mm; ML, ±0.30 mm; DV, −2.20 mm; injection 3: AP, +1.70 mm; ML, ±0.30 mm; DV, −2.00 mm. Concurrently, rAAV2/9-DIO-hSyn-GCaMP6m-WPRE-pA (5.54 × 10^12^ vg/ml; Brain VTA Co. Ltd.) was injected bilaterally into the PVN. In a separate parallel experiment for control labeling, rAAV2/R-EF1a-DIO-EGFP (5 × 10^12^ vg/ml; Brain VTA Co. Ltd.) was injected into the PrL and rAAV2/R-EF1a-DIO-mCherry (5 × 10^12^ vg/ml; Brain VTA Co. Ltd.) into the CA2 using the same stereotactic coordinates, while OXT-Cre was expressed in the ipsilateral PVN.

For in vivo photometry recordings, a Cre-dependent GCaMP6m virus was mixed with an OXT-promoter-driven Cre construct at a volume ratio of 3:1 (GCaMP6m:OXT-Cre), calculated on the basis of viral genome titers to achieve the desired functional expression. Similarly, for circuit-specific optogenetic manipulations involving PVN^OXT^ neurons, viral mixtures (hChR2, eNpHR, Tettoxlc, jGCaMP7b, or control) were combined with OXT-Cre at a 2:1 titer-adjusted ratio to ensure selective expression in OXT-producing neurons.

Two weeks after viral injection, optic fibers were implanted and secured with dental cement. For optogenetic manipulations, a 200-μm optic fiber (numerical aperture, 0.37; Inper, China) was implanted 0.4 mm above the injection site. For fiber photometry, the optic fiber was placed 0.2 mm above the PVN, CA2, or PrL.

### Cannula implantation and intra-PrL infusion

Cannula implantation and local drug administration were performed following previously established protocols with minor modifications [53]. Mice were anesthetized with 1% isoflurane and secured in a stereotaxic frame (E07370-005, RWD). Body temperature was maintained using a thermostatically controlled heating pad. Stainless-steel guide cannulas (outer diameter, 0.4 mm; inner diameter, 0.2 mm) were bilaterally implanted above the PrL with a 20° lateral tilt. Target coordinates relative to bregma were as follows: AP, +1.95 mm; ML, ±1.20 mm; and DV −1.75 mm from the pial surface. To maintain patency, each guide cannula was occluded with a solid-stainless-steel wire (diameter: 0.18 mm) that extended 0.2 mm beyond the cannula tip. The assembly was fixed to the skull using dental cement to ensure long-term stability. Animals were allowed to recover for a minimum of 7 d prior to experimental use.

For local drug infusion, mice were lightly anesthetized with 1% isoflurane. Either saline or the selective OXTR antagonist L-368,899 (MedChemExpress, NJ, USA; 1.25 mM in 500 nl per hemisphere) [54] was delivered directly into the PrL via an injection cannula (outer diameter, 0.18 mm; inner diameter, 0.09 mm), extending 0.25 mm beyond the guide. The infusion was carried out at a rate of 150 nl/min, followed by a 6-min retention period to facilitate tissue diffusion. On the basis of previous reports [55,56], the pharmacological effect of L-368,899 is expected to commence within 30 min postinfusion and persist for approximately 2–4 h. After the completion of behavioral testing, cannula placement and injection accuracy were verified by immobilizing the mice and examining the implantation sites.

### Fiber photometry recordings and data analysis

Neuronal activity and neurochemical dynamics were monitored using a fiber photometry system (Thinker Tech, Nanjing, China), which enabled simultaneous detection of calcium-dependent (e.g., GCaMP) and calcium-independent (e.g., GRAB_OXT1.8_) fluorescence signals. The system utilized a dual-wavelength approach: 470-nm excitation was used to elicit signal-dependent fluorescence, while the 405-nm reference channel served to correct for motion-related artifacts. Light intensity at the fiber tip was calibrated to 10 to 20 μW. Signal acquisition was conducted at a sampling rate of 30 Hz using the fiber photometry system (ThinkerTech, Nanjing, China). Raw fluorescence traces were exported and analyzed in MATLAB (R2023b, MathWorks) via custom-written scripts. Photobleaching and baseline drift were first corrected. Fluorescence data were then transformed into *z*-scores based on the following formula: *z*-score = [*F* − mean(*F*_baseline_)]/SD (*F*_baseline_), where *F*_baseline_ was defined as the fluorescence during a 2-min pretest baseline period [6].

Behavioral annotations were synchronized to the photometry recordings. Social interaction events were manually scored from video recordings by experimenters blinded to the fluorescence data. The start of an interaction bout was marked by direct physical contact (e.g., nose, head, or forelimb touching the wire cup). Consecutive bouts separated by < 0.5 s were merged, and bouts shorter than 1 s were excluded. For each valid interaction, timestamps were used to extract aligned *z*-score segments. Within each interaction episode, the maximum *z*-score observed during a 2-s window was quantified. The area under the curve (AUC) for the interaction period was computed using MATLAB’s trapz function. In addition, the mean *z*-score change was calculated by subtracting the median *z*-score within the 2-s prebout window from the interaction peak value (see Fig. 5H and J).

### Sleep deprivation

SD were induced using an automated disruption system consisting of a polyvinyl chloride cylindrical chamber (40-cm height × 40-cm diameter) equipped with a motor-driven horizontal bar traversing the enclosure. Mice were individually placed in the apparatus with fresh bedding, food, and water for at least 24 h prior to the start of experiments to allow habituation. Following each SD or control session, animals were returned to separate home cages maintained under identical housing conditions. For chronic SD, the bar was rotated continuously at a speed of 3 rpm for 4 h per day across 5 consecutive days during the light phase (11:00 to 15:00). In the acute SD protocol, the same rotational speed was applied over a continuous 12-h period (07:00 to 19:00) within a single light cycle. To minimize adaptation and prevent microsleep episodes, the rotation direction was periodically reversed in a randomized manner. Throughout all disruption sessions, a trained experimenter visually monitored the apparatus to confirm uninterrupted bar motion and ensure that mice did not adopt alternative postures or behaviors that would permit sleep.

### Optogenetic stimulation

For light delivery, mice were tethered to an optical fiber patch cord (4 m, 200-μm core diameter; Inper) connected to an intelligent optogenetic control system (ThinkerTech, China). A secondary patch cord linked the rotary joint to a light source capable of emitting either 473-nm blue light, 593-nm yellow light, or 635-nm red light.

For optogenetic activation, a 473-nm diode-pumped solid-state laser was used to deliver light pulses at a power of ~5 mW (measured at the fiber tip). The high-frequency stimulation protocol consisted of five 1-s trains of 100-Hz pulses (2-ms pulse width per pulse), with 15-s intervals between consecutive trains. For low-frequency stimulation, 4-Hz pulses (2 ms pulse width) were delivered in four 25-s bouts, again at ~5 mW, with 15-s interbout intervals. For optogenetic inhibition, a 593-nm laser was used to deliver continuous light at a power of 8 to 12 mW at the fiber tip during the specified behavioral or physiological windows. Both paradigms targeted PVN^OXT^ neurons to compare the frequency-dependent modulation of circuit activity and behavioral outcomes.

### Chemogenetic inhibition by clozapine *N*-oxide injections

On each test day, clozapine *N*-oxide (CNO; catalog no. 190924, Brain VTA Co. Ltd.) was freshly prepared at 0.05 mg/ml in 1% dimethyl sulfoxide. Animals received an intraperitoneal injection of either CNO (1 mg/kg) or saline 30 min before behavioral assessment.

### In vitro electrophysiology

On the third day following high-frequency (100-Hz) photostimulation in sleep-deprived mice, coronal brain slices containing the PVN were prepared for electrophysiological recordings, following procedures adapted from previously published protocols [57]. Mice were deeply anesthetized with sodium pentobarbital and transcardially perfused with ice-cold, oxygenated-sucrose-based artificial cerebrospinal fluid (ACSF; pH 7.3, saturated with 95% O_2_ and 5% CO_2_). The composition of the sucrose ACSF was as follows: 213 mM sucrose, 2.5 mM KCl, 26 mM NaHCO_3_, 10 mM glucose, 3 mM MgSO_4_, 1.25 mM NaH_2_PO_4_, 2 mM sodium pyruvate, 0.4 mM ascorbic acid, and 0.1 mM CaCl_2_.

Brains were rapidly extracted and sectioned into 320-μm coronal slices using a vibrating microtome (VT1200, Leica, Germany) under ice-cold conditions. Sections containing the PVN were immediately transferred into standard recording ACSF containing 126 mM NaCl, 25 mM glucose, 2.5 mM KCl, 2 mM CaCl_2_, 1.25 mM NaH_2_PO_4_, 26 mM NaHCO_3_, and 1.0 mM MgSO_4_. Slices were initially incubated at 32 °C for 30 min to allow recovery and then maintained at room temperature for an additional 30 min before commencing electrophysiological procedures. During recordings, slices were continuously superfused with warmed ACSF (30 to 32 °C) at a flow rate of 2 ml/min within a submerged recording chamber. The PVN region was localized using low-magnification epifluorescence imaging, while target neurons were visualized under high-magnification fluorescence microscopy. Patch-clamp electrodes (resistance, 4 to 6 MΩ) were filled with an internal pipette solution composed of 105 mM potassium gluconate, 30 mM KCl, 4 mM Mg-adenosine triphosphate, 10 mM phosphocreatine, 0.3 mM EGTA, 0.3 mM Na-guanosine triphosphate, and 10 mM Hepes (adjusted to pH 7.3, 285 to 300 mOsm). Both cell-attached and whole-cell recordings were performed in current–clamp and voltage–clamp modes (holding potential −70 mV), using a MultiClamp 700B amplifier (Axon Instruments). Electrophysiological signals were low-pass-filtered at 4 kHz and digitized at 10 kHz with a Digidata 1440A digitizer (Axon Instruments). Data acquisition and offline analysis were conducted using pClamp 10.3 software (Axon Instruments).

### Behavioral tests

All behavioral experiments were conducted during the animals’ active (dark) phase, between 7:00 PM and 12:00 PM, in a dedicated testing room illuminated by dim red light to minimize visual interference. Prior to testing, mice were acclimated to the environment for a minimum of 3 h. Behavioral performance was captured using an automated video-tracking system (VisuTrack, Shanghai, China), and subsequent analyses were performed offline by experimenters blinded to treatment conditions. Videos were acquired with a TC420HD camera at a resolution of 1,280 × 720 pixels and a frame rate of 15 frames/s. All videos were saved in AVI format for subsequent analysis. These parameters ensure sufficient spatial and temporal resolution for reliable automated behavioral quantification. Social interaction was defined as the animal actively orienting toward and making contact with the wire cage using its nose, vibrissae, or forepaws within a 0 to 3 cm range. Instances where the mouse simply passed by or faced away from the cage were not considered social interaction. Animals exhibiting low exploratory behavior, defined as an average object investigation time of less than 10 s, were excluded from further analysis. In total, 5 mice were removed from the dataset based on this criterion due to insufficient engagement with the test apparatus.

### Two-choice social memory test

This test was performed as previously described [46]. The social memory assay was conducted in a rectangular open field arena (54 cm × 26 cm). In the first phase (trial 1; habituation), the subject mouse was allowed to freely explore the arena for 5 min in the presence of 2 empty wire cages placed at opposite ends. In the subsequent phase (trial 2; social memory encoding), 2 unfamiliar stimulus mice (designated S1 and S2) were individually confined to the cages, and the subject mouse was reintroduced into the arena for another 5 min of exploration. After a 30-min intertrial interval, the final phase (trial 3; social memory retrieval) was performed. One of the previously encountered stimulus mice (S1 or S2) was replaced with a novel conspecific (N), while the other remained unchanged (designated as S). The subject mouse was again allowed to explore the arena for 5 min: Discrimination index N = (time exploring mouse N − time exploring mouse S) / (time exploring mouse N + time exploring mouse S); Discrimination index F = (time exploring mouse S1 or S2 in in trial 2 − time exploring mouse S in trial 3) / (time exploring mouse S1 or S2 in in trial 2 + time exploring mouse S in trial 3). Normal social memory is demonstrated by the following: (a) An increased exploration time of the novel mouse (N) compared to the familiar stimulus mouse (S1 or S2) during trial 3, measured by the discrimination index N; and (b) a reduced exploration time of the familiar stimulus mouse (S1 or S2) in trial 3 relative to trial 2, measured by the discrimination index F (see Fig. 1A).

### Five-trial social memory test

This test was adapted from previous work [56,58]. The subject mouse was habituated for 5 min to a rectangular arena (54 cm × 26 cm) with one empty cage in opposite sides. Subject mice were presented with a stimulus mouse for 4 successive 2-min trials, separated by 10-min intertrial intervals. On the fifth trial, another novel stimulus animal was presented (see Fig. 2J).

### Social interaction test

The behavioral protocol was adapted from previously published methods [27]. The testing arena’s central region was illuminated by halogen lamps set to approximately 200 lux, positioned 200 cm above the floor. Prior to testing, mice were acclimated to the experimental room environment for 30 min to reduce stress and habituate to ambient conditions. Subsequently, each mouse was placed in an open-field Plexiglas chamber (50 cm × 50 cm × 50 cm) containing an empty wire cage positioned at one end. Spontaneous locomotion and baseline exploratory activity were recorded continuously for 5 min using an automated tracking system. After this baseline session, the mouse was briefly removed, and the arena was thoroughly disinfected with 75% ethanol to eliminate olfactory cues. Next, a novel social stimulus mouse (C57 strain) was introduced into the previously empty cage, and exploratory behavior directed toward this social target was recorded for an additional 5 min. The primary metric for analysis was the duration spent in the predefined interaction zone near the cage. Social engagement was quantified by calculating the interaction ratio, defined as the percentage of time spent interacting with the novel C57 mouse relative to the time spent exploring the empty cage, using the formula: [100 × (interaction time, “novel C57”) / (interaction time, “empty cage”)] (see Fig. S1A).

### Three-chamber social interaction

The behavioral paradigm was performed following previously established protocols [6]. Briefly, the apparatus consisted of 3 interconnected chambers: 2 side compartments measuring 26 cm × 23 cm each, and a central chamber measuring 11 cm × 23 cm, with doors allowing free access between chambers. Initially, the subject mouse was placed in the central chamber and permitted to explore all 3 compartments freely for 10 min in the absence of any social stimuli (habituation phase). Subsequently, the mouse was returned to the central chamber, and the connecting doors were temporarily blocked using white plastic covers. An empty wire cage (10 cm in diameter; designated “E”) was positioned randomly in one of the side chambers, while an identical cage housing a novel conspecific (stimulus mouse S1) was placed in the opposite side chamber. Following removal of the door covers, the subject was allowed to freely explore and interact with either the empty cage or the social stimulus (S1) for a 10-min session (trial 1). Upon completion, a second unfamiliar mouse (S2) was introduced into the previously empty cage, and the subject was again granted 10 min to explore both social targets (trial 2). The locations of the empty cage and stimulus mice (S1 and S2) were counterbalanced across subjects to prevent side bias. Social preference was quantified using 2 indices: Sociability index = [(time spent exploring mouse S1) − (time spent exploring empty cage)] / [(time spent exploring mouse S1) + (time spent exploring empty cage)]; Social novelty index = [(time spent exploring mouse S2) − (time spent exploring mouse S1)] / [(time spent exploring mouse S2) + (time spent exploring mouse S1)] (see Fig. S1D).

### Two-trial social memory test

This behavioral assay was conducted according to previously established protocols [27]. Briefly, during trial 1, the subject mouse was allowed to freely explore an arena containing one empty cage and another cage housing a novel mouse for 5 min. Following this, the mouse was isolated for 30 min within the same arena, which now contained 2 empty cages positioned on opposite sides (trial 2). In trial 3, the subject mouse was reintroduced and exposed simultaneously to the familiar stimulus mouse (S1) from trial 1 alongside a novel conspecific (N). Social memory was evaluated by calculating the discrimination index as the ratio of time spent investigating the novel mouse over the time spent exploring the familiar mouse (see Fig. S9A).

### Immunohistochemistry

Mice were deeply anesthetized with sodium pentobarbital and transcardially perfused via the left ventricle with saline, followed by 4% paraformaldehyde (PFA) solution. Extracted brains were postfixed in 4% PFA at 4 °C for 12 h and then transferred to a 30% sucrose solution in phosphate-buffered saline (PBS) for cryoprotection until they sank. Coronal brain sections (40 μm in thickness) were cut using a freezing microtome (CM1950, Leica, Germany) maintained at −23 °C.

For immunostaining, free-floating sections were first incubated in blocking buffer (P0260, Beo Tianmei, China) for 40 min at room temperature to reduce nonspecific binding. Subsequently, sections were incubated overnight at 4 °C with the primary antibody against OXT (EPR20973, Abcam, USA; 1:500) diluted in antibody diluent (P0262, Beo Tianmei, China). After thorough washing with PBS, sections were then incubated with the corresponding fluorescent secondary antibody (donkey anti-rabbit Alexa Fluor 546, GB21301, Servicebio; 1:800) for visualization.

To assess the colocalization between endogenous OXT and AAV-driven GCaMP6m expression, we performed immunohistochemistry on 40-μm brain sections and acquired high-resolution images using a confocal microscope (SP8, Leica). For each mouse, ×20 magnification fields were selected for analysis. Cell counting was conducted using ImageJ software, and all GCaMP6m-expressing cells within the entire PVN region were quantified.

To ensure accurate localization and delineation of the PVN, we relied on distinct anatomical landmarks, most notably the third ventricle, and compared each section with corresponding levels in the mouse brain in stereotaxic coordinates atlas. This approach allowed us to reliably identify PVN boundaries despite its complex anatomical location. We confirmed that the analysis encompassed the full PVN in each section. Cells were identified on the basis of colocalization with 4′,6-diamidino-2-phenylindole (DAPI) nuclear staining. Although occasional GCaMP6m-positive cells were observed near the periphery of the PVN, our analysis was restricted to cells within anatomically defined PVN boundaries based on stereotaxic coordinates and landmark structures.

To confirm viral expression and verify the placement of the optic fiber, mouse brains were processed as previously described. Coronal sections of 40 μm in thickness were obtained throughout the entire brain. Sections containing the fiber tract were washed 3 times with PBS and subsequently stained with DAPI for 20 min at room temperature to visualize cell nuclei. Only animals demonstrating viral expression restricted to the targeted brain region, with minimal off-target spread, and whose fiber optic tips were positioned directly above the central area of viral expression were included in the final dataset. The location of the fiber tip was determined by identifying the ventral-most boundary of the fiber tract. It should be noted that dashed lines representing the fiber tract trajectory are estimations and may not precisely correspond to the exact lowest point of the fiber tip.

Digital images were processed using ImageJ software to minimally adjust brightness and contrast. After completing the experiments, we assigned an ID to each mouse and matched it with the corresponding experimental results. Regarding viral expression sites, brain regions such as CA2 and PrL were identified with reference to the Brain Atlas, which allowed for relatively clear delineation. Mice with incorrect injection locations or poor viral expression were excluded from the analysis.

### Statistics

Data are expressed as means ± SEM. Differences in social memory indices between groups were analyzed using 2-way analysis of variance (ANOVA). Paired 2-tailed Student’s *t* tests were applied to compare OXT neurotransmitter levels and social memory indices before and after SD. For comparisons between 2 independent groups, unpaired 2-tailed Student’s *t* tests were used. *P* < 0.05 was considered statistically significant. Post hoc significance values were set as *P < 0.05, **P < 0.01, and ***P <0.001. The data were analyzed, and figures were drawn and adjusted using GraphPad Prism 10.0 software.

## Ethical Approval

All experimental protocols were approved by the Experimental Animal Ethics Committee of the Zhongnan Hospital, Wuhan University (license identification number: ZN2023198).

## Data Availability

Data supporting the results of this study are available from the corresponding author.
