# Supplementary material for: Decreased Oxytocin Mediates PVN–CA2 and PVN–PrL in Sleep Deprivation-Induced Social Memory Deficits
Source: Research (Wash D C). 2026 Feb 6;9:1076. doi: 10.34133/research.1076 (PMC12877408; doi:10.34133/research.1076)
Supplement: Supplementary 1 — Figs. S1 to S10 [file research.1076.f1.zip › Supplemental Material.docx]

**Oxytocin mediates resilience to** **social memory impairment caused by sleep deprivation**

Yanchao Liu et al. Email: yanchao_liu@whu,edu.cn

Corresponding author. Email: [linlinbi2016@whu.edu.cn](mailto:linlinbi2016@whu.edu.cn) (L.-L.B.); xuhaibo@whu.edu.cn (H.-B.X.)

**Supplementary figures and legends**

**
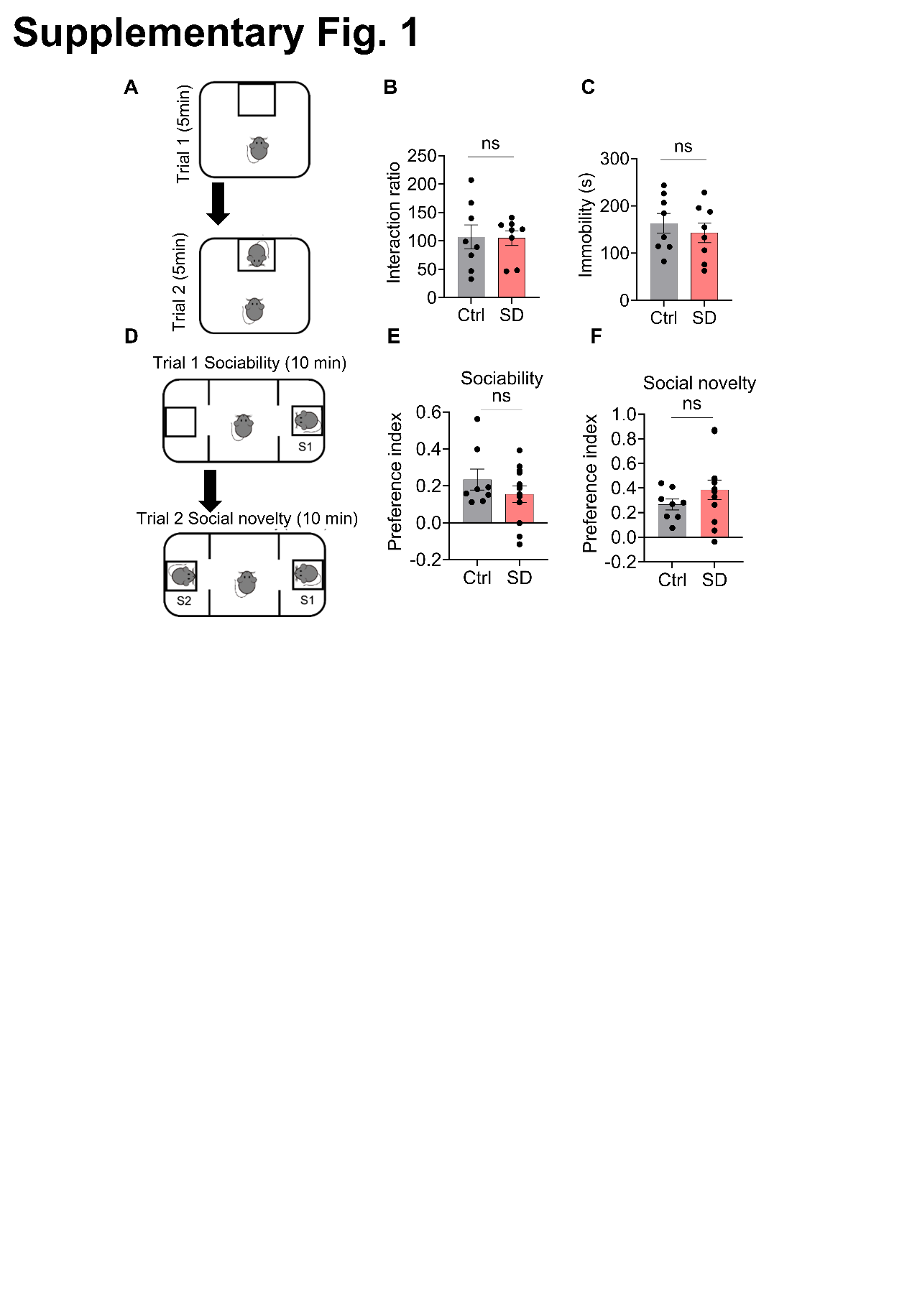
**

**Supplementary Fig.1 Chronic SD did not impair social exploration and social preference.**

**A.** Schema of the social interaction (SI) test.

**B and C.** Chronic SD did not impair social exploration ability. Unpaired t-test, N=8 mice per group.

**D.** Schema of the three-chamber social interaction test.

**E and F.** Chronic SD did not impair social preference. Unpaired t-test, N=8 mice in Ctrl group, N=12 mice in SD group.

Data are presented as mean ± SEM, ns, p＞0.05.


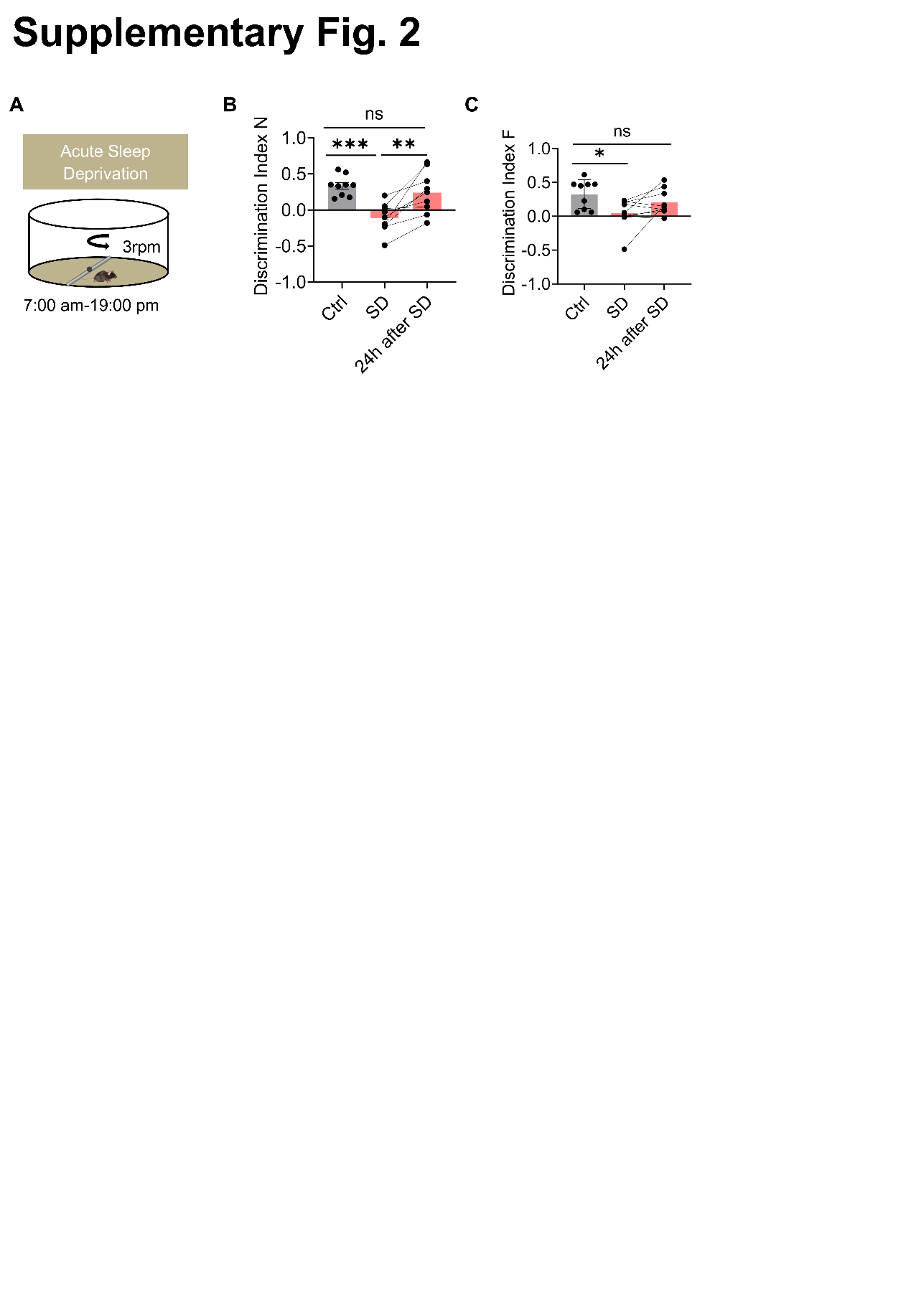


**Supplementary Fig.2 Acute SD caused transient social memory deficits.**

**A.** Acute SD protocol.

**B.** The social memory of all mice was detected using the “two-choice social memory test”. Acute SD mice spent less time exploring the novel mouse (N), reflected by a lower discrimination index N. But the index N rebounded after 24 hours recovery sleep, showing no significant difference from the control group. Unpaired t-test (Ctrl *vs.* SD) and Paired T-test (SD *vs.* 24h after SD), N=9 mice in Ctrl group, N=7 mice in acute SD group.

**C.** Acute SD mice spent more time exploring familiar mouse (S) with a lower discrimination index F. However, the index also rebounded after 24 hours of recovery sleep, showing no significant difference from the control group. Unpaired t-test (Ctrl *vs.* SD) and Paired t-test (SD *vs.* 24h after SD), N=9 in Ctrl group, N=7 mice in acute SD group.

Data are presented as mean ± SEM, ns, p＞0.05, *p<0.05, **p<0.01, ***p<0.001.


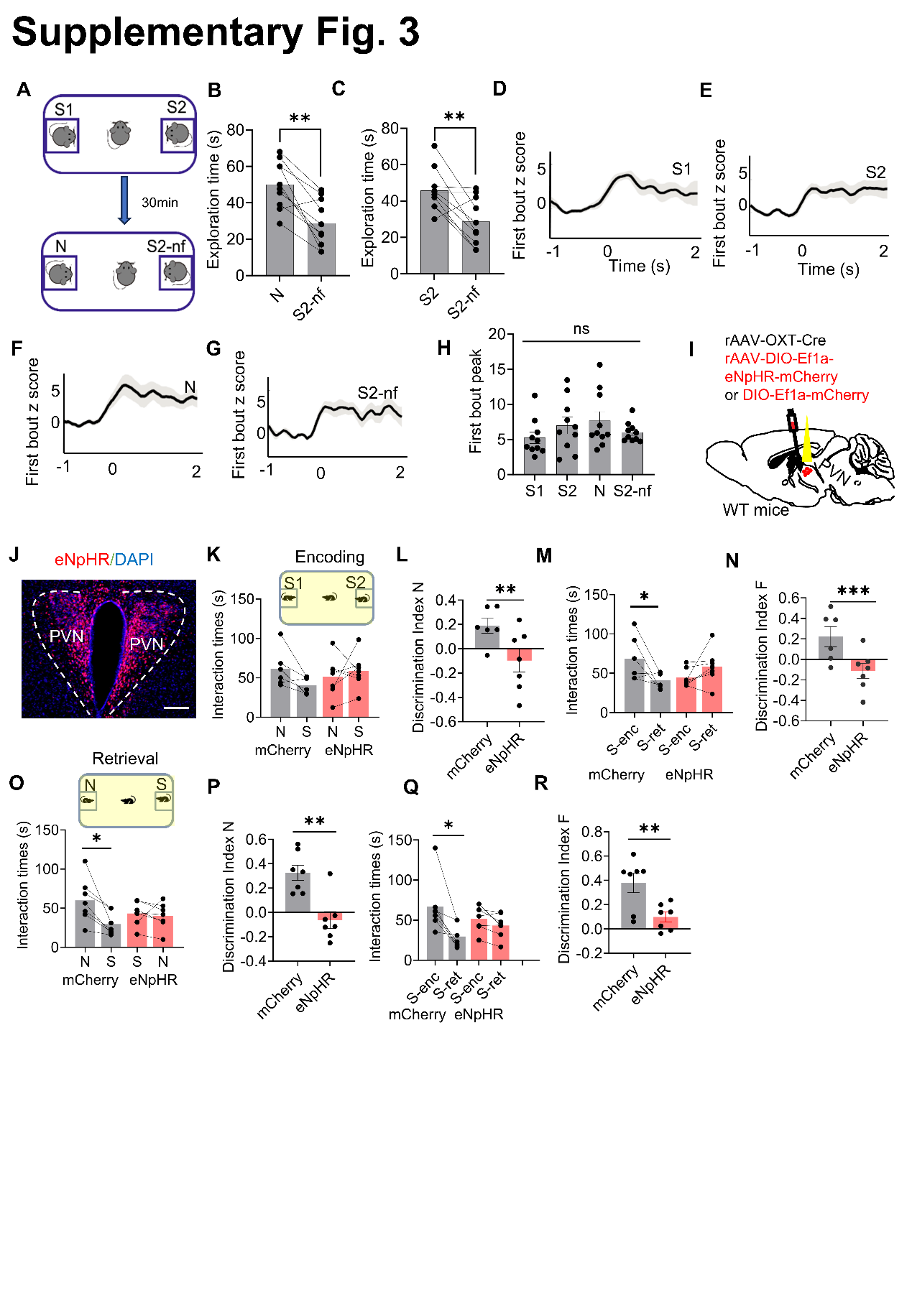


**Supplementary Fig.3 PVN^OXT^ neurons are essential for regulating social memory behavior.**

**A.** Two-choice social memory test. During the encoding phase, subject mice explored two novel mice (S1 and S2) for 5 minutes, followed by a 30 min intertrial interval. In the retrieval trial, one stimulus mouse (S1) was replaced by a third novel mouse (N), while the subject mouse explored the now-familiar mouse (S2-nf) and N. Mouse replaced (S1 or S2) was chosen randomly.

**B and C.** Subject mice spent more time exploring the novel mouse (N) than exploring the now-familiar mouse (S2-nf) during retrieval. Paired t-test, N=10 mice per group.

**c.** Exploration time of S2-nf during retrieval was shorter compared to S2 in the encoding trial. Paired T-test, N=10 mice per group.

**D-G.** Representative traces of Ca^2+^ signals of PVN^OXT^ from subject mice (N = 10 mice). Peri-event plots display averaged fluorescence, with curves and shaded regions indicating the mean ± SEM.

**H.** First bout peak Ca^2+^ signals of PVN^OXT^ during exploration bouts in encoding (S1, S2) and retrieval (S2-nf, N) trials. No differences were found among the four conditions. one-way ANOVA, N=10 mice per group.

**I** Diagram of bilateral eNpHR virus expression using a mixture of rAAV-OXT-Cre and rAAV-CAG-DIO-eNpHR-mCherry in the PVN, with fiber implantation above the PVN.

**J.** Histological confirmation of eNpHR expression in the PVN^OXT^ neurons. Scale bar: 200 µm.

**K-R.** Shining yellow light on PVN^OXT^ during the encording phase in the two-choice social memory test (k-n, trial 2, eNpHR N=7 mice, mCherry=6 mice) or during the retrieval phase (o-r, trial 3, N=7 mice per group) impaired social memory of animals expressing eNpHR in PVN^OXT^ neurons, relative to the control group expressing mCherry. N: Exploration time with a novel mouse during the retrieval phase; S: Exploration time with a familiar mouse (S1/S2) during the retrieval phase; S-enc: Exploration time with mouse S1/S2 during the encoding phase; S-ret: Exploration time mouse S (S1/S2) during the retrieval phase. Paired or unpaired t-test was used.

Data are presented as mean ± SEM, *p<0.05, **p<0.01, ***p<0.001.


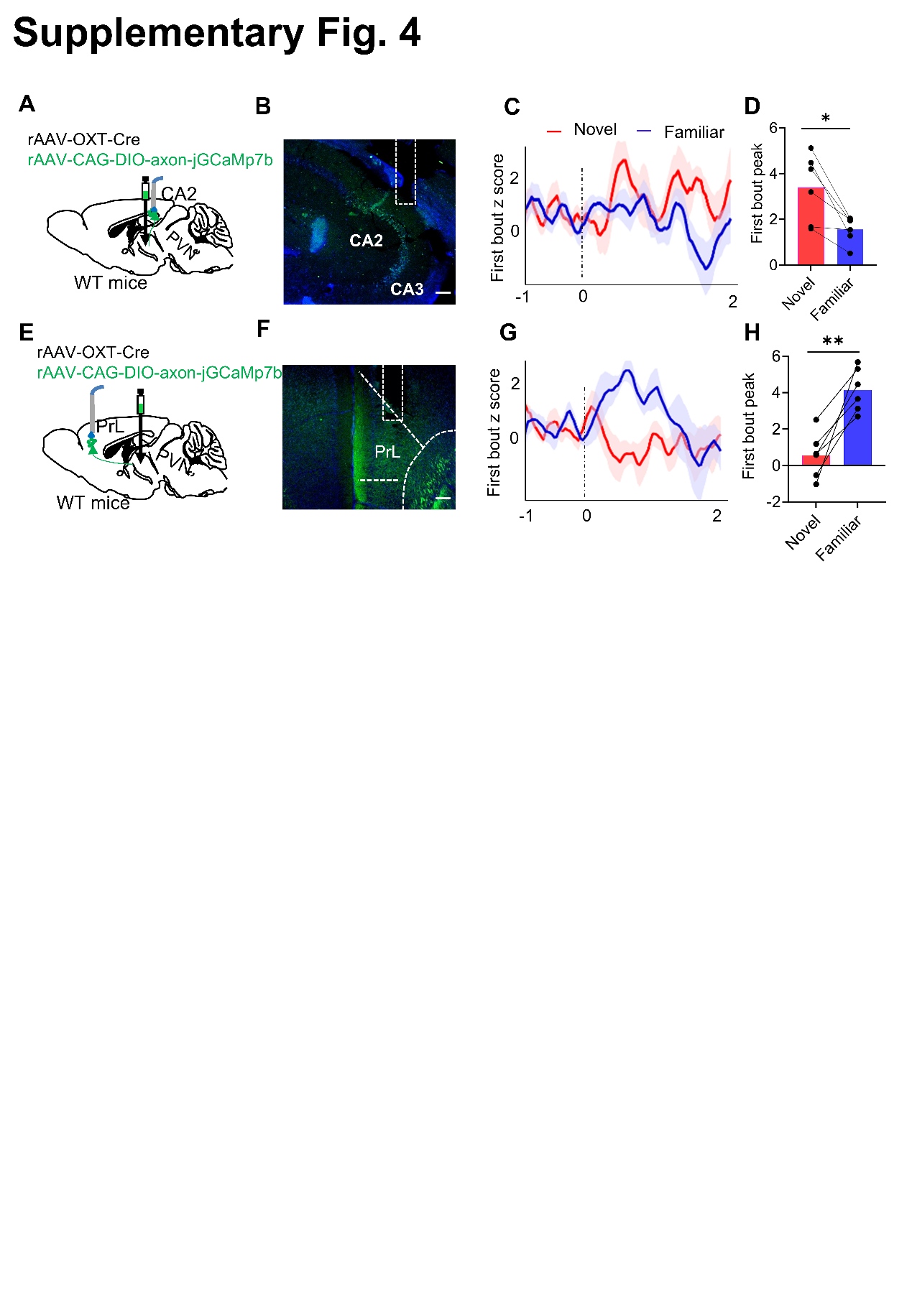


**Supplementary Fig.4 PVN^OXT^-CA2 and PVN^OXT^-PrL terminal activation differs during social exploration**

**A**. Diagram of unilateral axon-jGCaMP7b expression in the PVN and fiber photometry in the CA2.

**B**. Histological confirmation of jGCaMP7b expression in the CA2. Scale bar: 500 µm.

**C and D** Ca^2+^ signal in axonal terminals of the PVN^CA2^ projection were elevated during the first interaction bouts with novel mice, while no change is observed in presence of a familiar mouse. Peri-event plots display averaged fluorescence, with curves and shaded regions indicating the mean ± SEM. Paired t-test, N=6 mice per group.

**E**. Diagram of unilateral axon-jGCaMP7b expression in the PVN and fiber photometry in the PrL.

**F.** Histological confirmation of jGCaMP7b expression in the PrL. Scale bar: 500 µm.

**G and H.** Ca^2+^ signals in axonal terminals of the PVN^Prl^ projection exhibited were elevated during the first interaction bouts with familiar mice, while no change is observed in presence of a novel mouse. Paired t-test, N=6 mice per group.


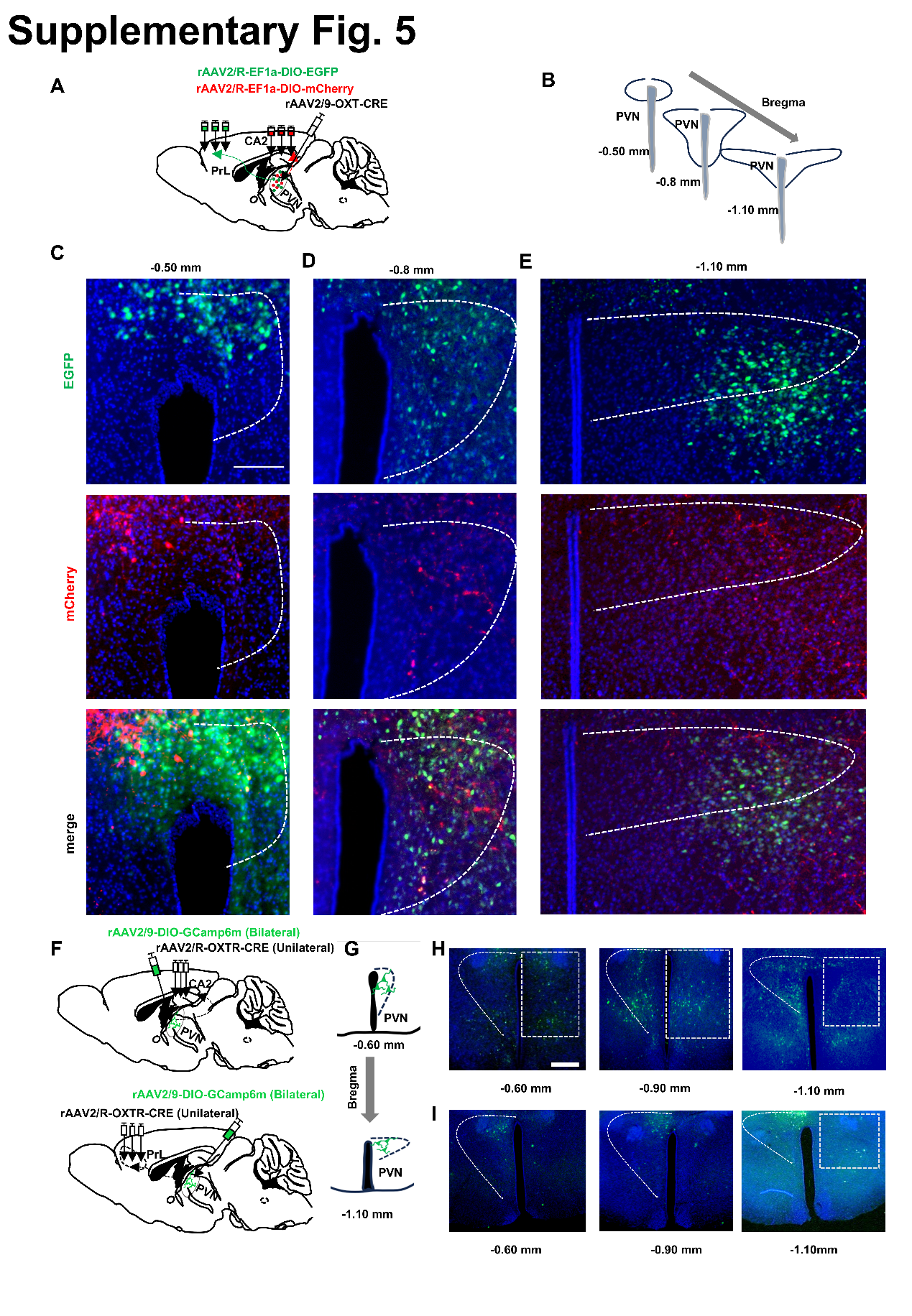


**Supplementary Fig. 5**

**A**. Schematic of unilateral injections of rAAV2/R-AAV-DIO-hSyn-EGFP into the PrL and rAAV2/R-AAV-DIO-hSyn-mCherry into the CA2 (N = 5 mice), together with ipsilateral injection of AAV-OXT-cre into the PVN.

**B.** Coronal sections were collected across three rostro-caudal levels for histological analysis.

**C–E.** OXT neurons projecting to CA2 (red) and those projecting to the PrL (green) show minimal overlap.

**F.** Schematic of unilateral rAAV2/R-OXTR-CRE injection into either the CA2 (N = 2 mice) or the PrL (N = 2 mice), combined with bilateral AAV-DIO-hSyn-GCaMP6m injection into the PVN.

**G.** Coronal sections prepared from anterior to posterior relative to bregma for histological observation.

**H–I.** Subtype classification of PVNOXT neurons projecting to CA2 (h) and PrL (i) (left curves). A subset of PVNOXT neurons project to the contralateral CA2 or PrL, and these neuronal populations are largely non-overlapping (right box).


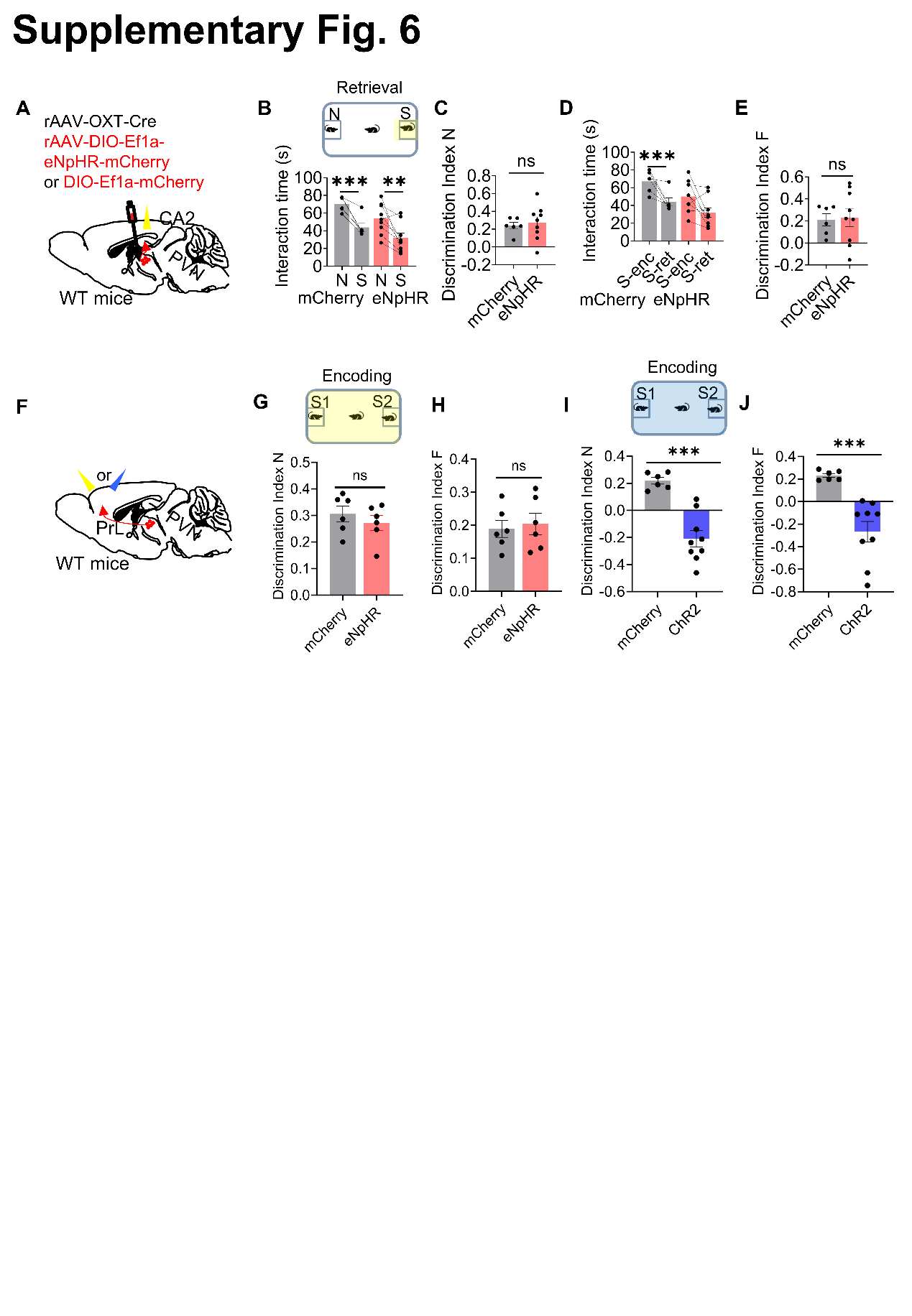


**Supplementary Fig. 6 PVN^OXT^-CA2 and PVN^OXT^-PrL pathway respectively regulated social memory encoding and retrieval.**

**A-E.** Diagram of eNpHR or mCherry virus injection in PVN^OXT^ neurons and fiber implantation above CA2 (a). Photoinhibition of PVN^OXT^-CA2 pathway during the retrieval phase (trial 2, eNpHR N=9 mice, mCherry N=6 mice) (b-e) in two-choice social memory test, did not impair social memory performance of animals expressing eNpHR in PVN^OXT^ relative to the control group expressing mCherry.

**F.** Diagram of photoactivation or photoinhibition of the PVN^OXT^-PrL pathway.

**G AND H.** Optogenetic inhibition of the PVN^OXT^-PrL pathway during encoding phase did not impair social memory. Unpaired t-test. N=6 mice per group.

**I AND J.** Optogenetic activation of the PVN^OXT^-PrL pathway during encoding phase impaired social memory. Unpaired t-test, N=6 mice in mCherry group, N=9 in ChR2 group. Data are presented as mean ± SEM, *p<0.05, **p<0.01, ***p<0.001.


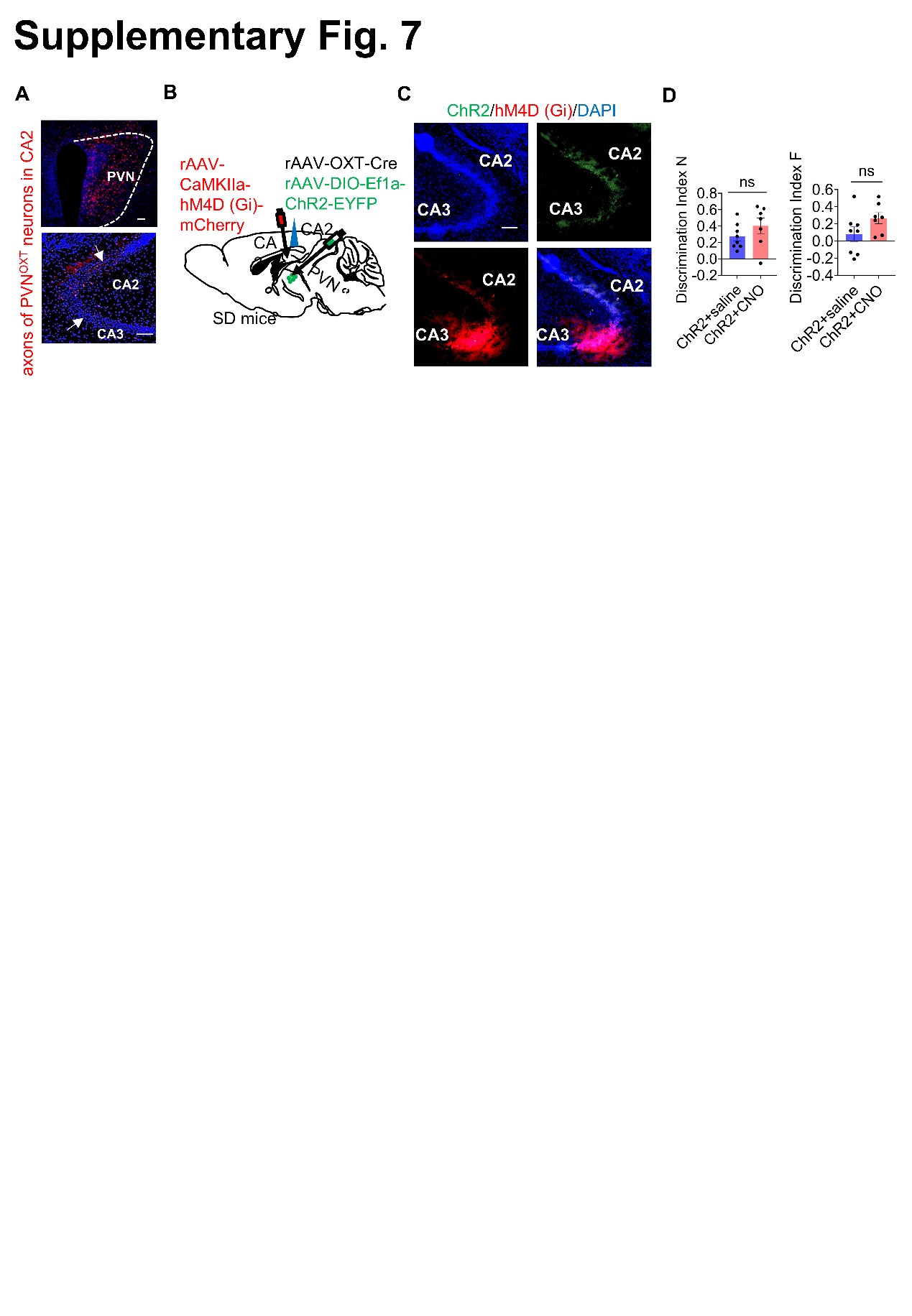


**Supplementary Fig. 7 Activation of the PVN^OXT^-CA3 pathway did not improve social memory deficits in SD mice.**

**A.** The CA3 also receives projections (white arrows) from the PVN^OXT^ neurons. Scale bars: 200 µm for PVN; 500 µm for CA2.

**B.** Diagram of unilateral hM4D virus (rAAV-CaMKIIa-hM4D (Gi)-mCherry) in CA3, unilateral mix virus (rAAV-OXT-Cre and rAAV-CAG-DIO-ChR2-mCherry) in PVN and fiber was implanted up CA2.

**C.** Histological confirmation of DAPI (blue), ChR2 (green) and hM4D (red) expression in CA3. Scale bar: 500 µm.

**D.** Activation of PVN^OXT^-CA2 during the social memory encoding stage in the two-choice social memory test improved social memory in SD mice despite CA3 chemogenetic inhibition. Unpaired t-test, N=8 mice in ChR2+saline group, and N=7 in ChR2+h4MDi group (one mouse was excluded because of fiber loss).

Data are presented as mean ± SEM, *p<0.05, **p<0.01, ***p<0.001.


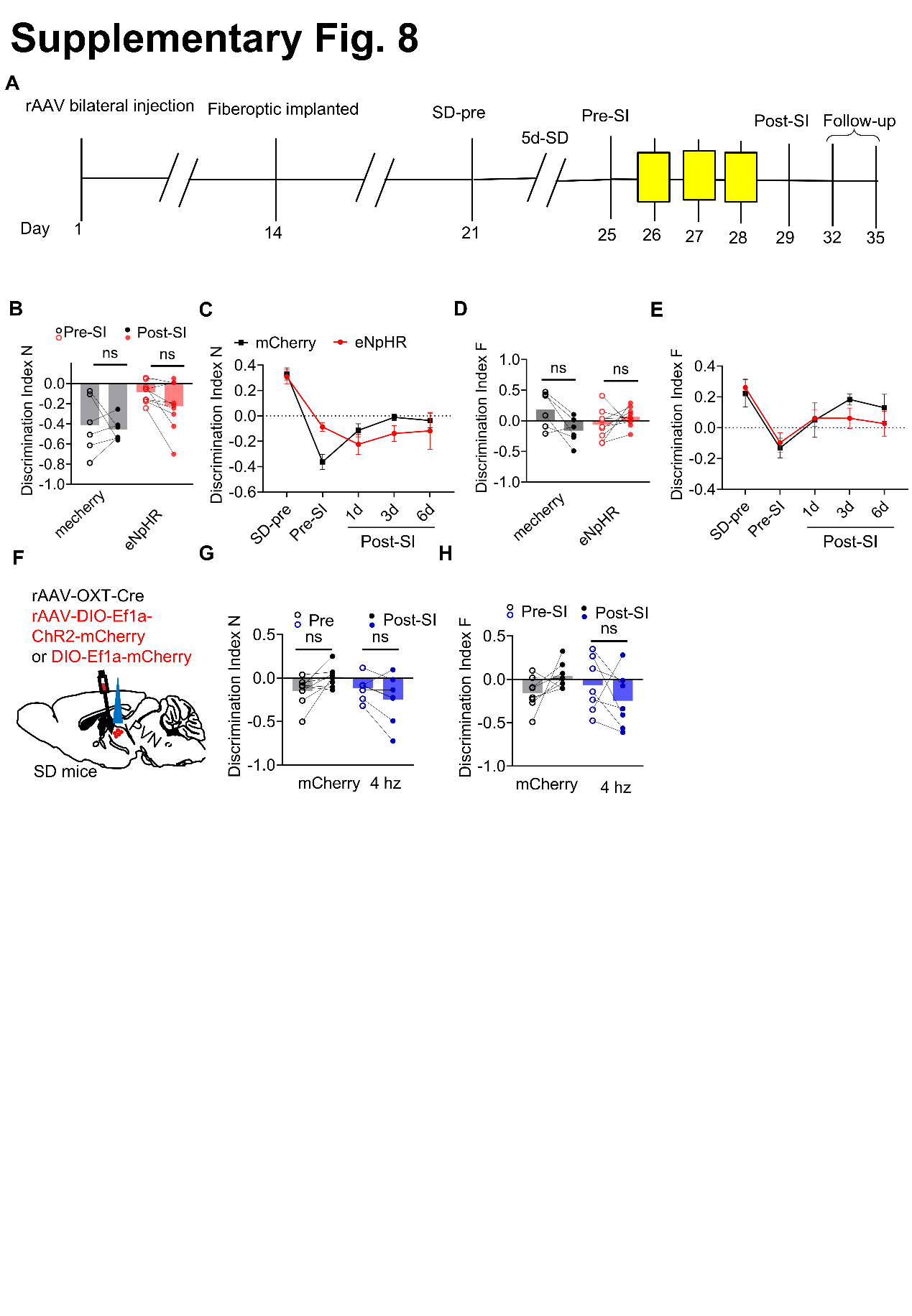


**Supplementary Fig. 8 Short-term hyperpolarization and low frequency depolarization of PVN^OXT^ neurons does not promote resilience of social memory.**

**A.** Experimental timeline in figure b-e. Pre-SI, before pulsed photo-inhibition; Post-SI, after pulsed photo-inhibition. Pulsed photo-inhibition light was described in the previous literature^26^ (7s × 10 with a 25 s interstimulus interval each day for three consecutive days).

**B-E.** Compared with control group (mCherry), short-term hyperpolarization treatment (eNpHR) did not improve the social memory deficits in SD mice in the two-choice social memory test. Unpaired t-test, N=6 mice per group.

**F.** Diagram of bilateral ChR2 virus expression by mix of rAAV-OXT-Cre and rAAV-CAG-DIO-ChR2-mCherry in PVN and fiber implantation above PVN.

**G AND H.** Shining 4Hz blue light (four bouts of 4Hz stimulation for 25s; ~5mW 473nm, 2ms pulse width; with 15s between bouts) on PVN^OXT^ did not improve SD-induced social memory impairments. Paired t-test, N=8 mice in mCherry group, N=7 mice in ChR2 group. Data are presented as mean ± SEM, *p<0.05, **p<0.01, ***p<0.001.


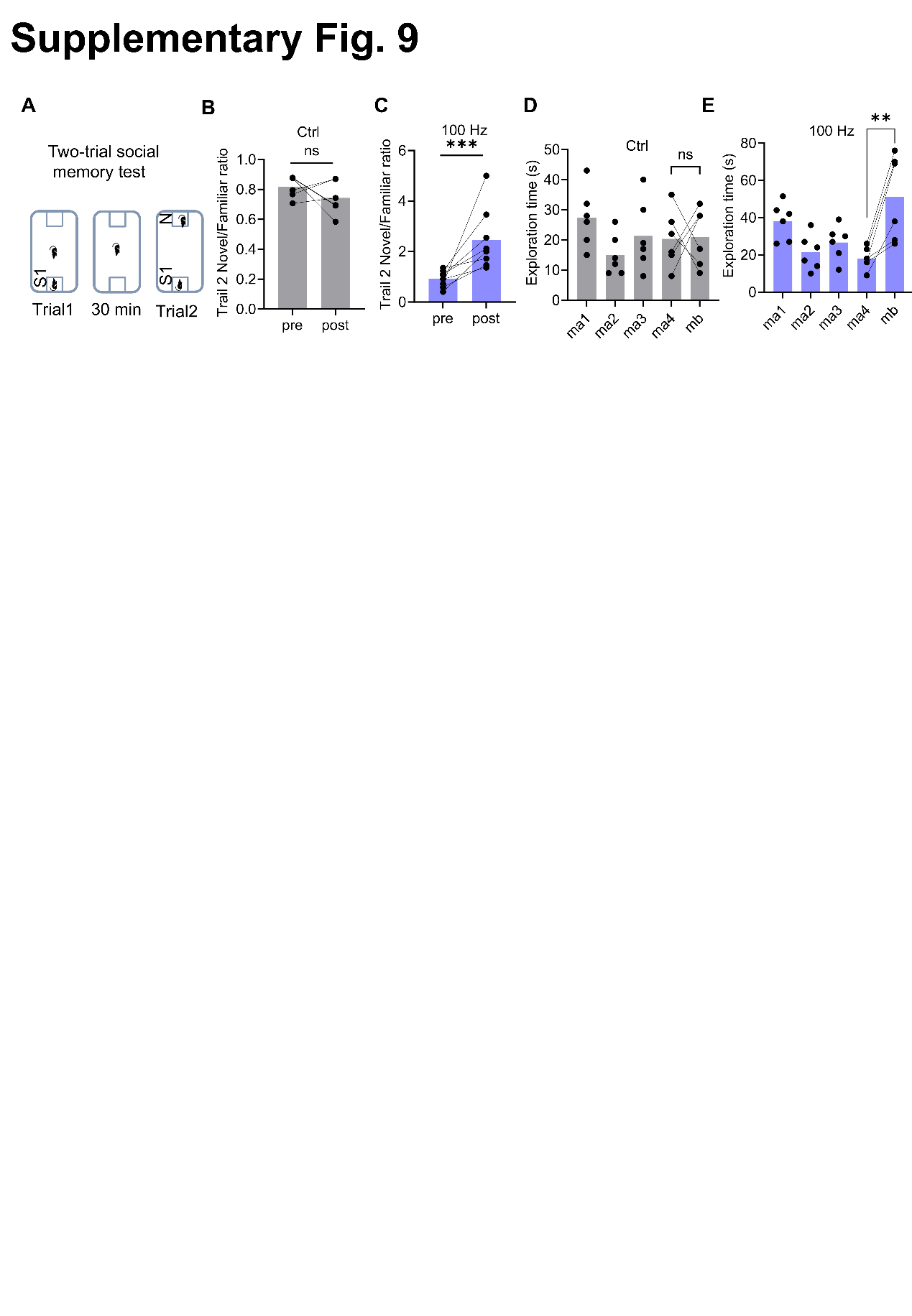


**Supplementary Fig.9 Social memory improvements after 100 Hz optogenetic activation were observed in two-trial social memory test and five-trial social memory test.**

**A.** Two-trial social memory test. Chronic SD mice (ctrl) spent more time exploring the mice S1, showing a lower ratio.

**B AND C.** Mice in the 100 Hz group spent more time exploring mouse N with a higher ratio compared to ctrl group. Unpaired t-test, N=6 mice per group.

**D AND E.** 100 Hz treatment significantly improved social memory impairment in SD mice in five-trial social memory test. Unpaired t-test, N=6 mice per group.

Data are presented as mean ± SEM, *p<0.05, **p<0.01, ***p<0.001.

**
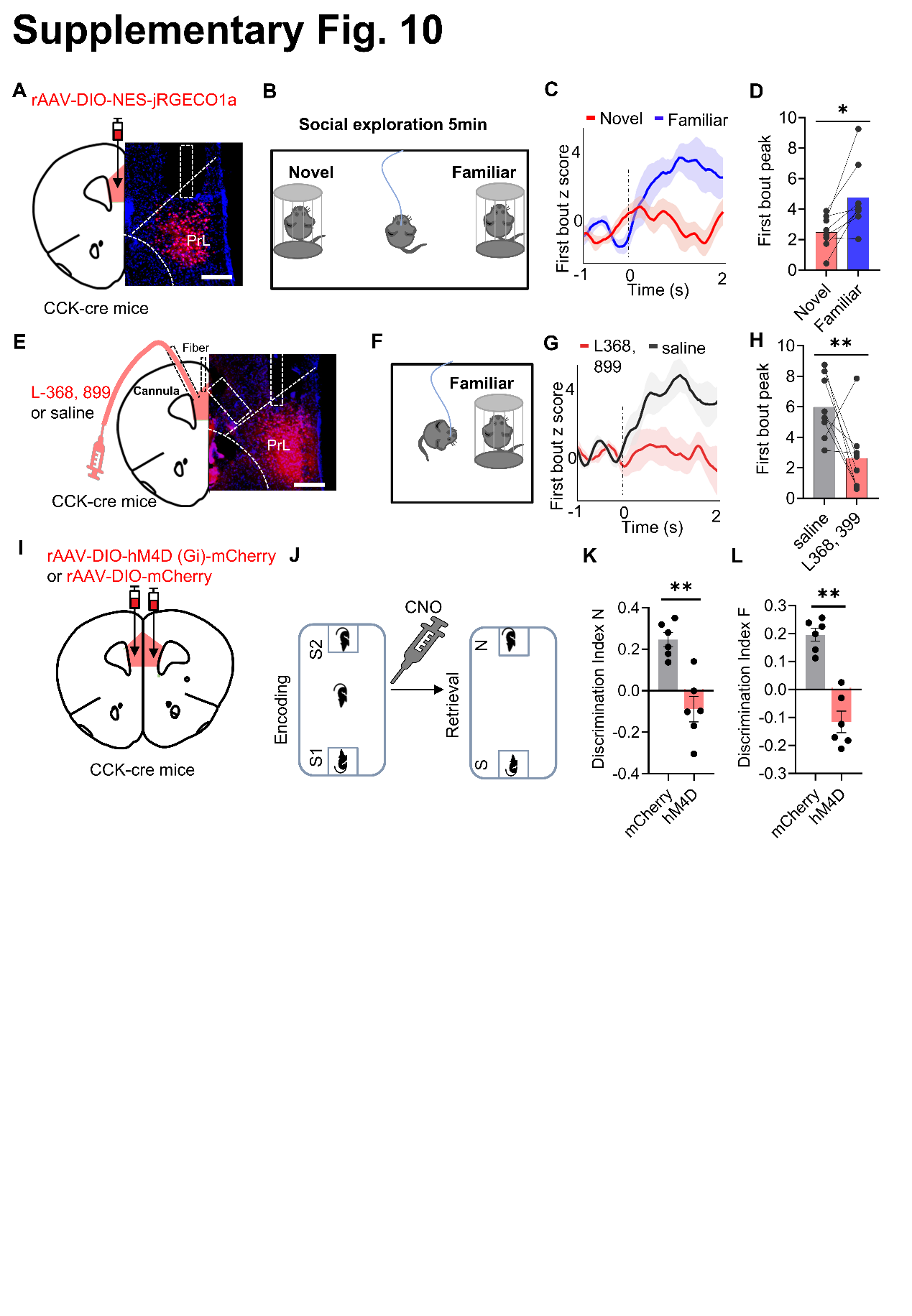
**

**Supplementary** **Fig. 10 CCK neurons in PrL are involved in OXT-regulated social memory retrieval.**

**A-D.** CCK neuron activity in the PrL increased during initial interactions with familiar mice but remained unchanged during interactions with novel mice.

**A.** Schematic showing unilateral rAAV-DIO-NES-jRGECO1a virus injection into the PrL of CCK-Cre mice and subsequent fiber photometry. Scale bar: 500 µm.

**B.** Ca^2+^ recordings from CCK neurons in the PrL during exploration of both familiar and novel mice, measured via fiber photometry.

**C-D.** Ca^2+^ signals in PrL CCK neurons increased during initial interactions with familiar mice, while no significant changes occurred during interactions with novel mice. Paired t-test, N = 8 mice per group.

**E-H.** After applying an OXT receptor antagonist in the PrL, CCK neurons failed to activate during the initial exploration of a familiar mouse.

**E.** Diagram showing cannula placement and fiber photometry setup in the PrL. Scale bar: 500 µm.

**F.** Ca^2+^ signals of CCK neurons were recorded during the initial interaction with a familiar mouse following local application of either an OXT receptor antagonist or saline.

**G AND H.** The peak Ca^2+^ activity of CCK neurons during initial interactions with a familiar mouse was significantly reduced following local application of the OXT receptor antagonist.

**I-L.** Bilateral chemogenetic inhibition of CCK neurons in the PrL during memory retrieval impaired social memory in mice.

**I.** Schematic showing bilateral rAAV-DIO-hM4D(Gi)-mCherry or rAAV-DIO-mCherry virus injections into the PrL of CCK-Cre mice. N = 6 mice per group.

**J.** Social memory was assessed using the “two-choice social memory test.” After encoding trial, mice received intraperitoneal CNO injections, and subsequent retrieval trials were conducted 30 minutes later.

**K-L.** Mice expressing hM4D showed reduced discrimination index N and F.

**Antibodies and virus**

| **REAGENT or RESOURCE** | SOURCE | IDENTIFIER |  |
| --- | --- | --- | --- |
| **Antibodies** |  |  |  |
| Alexa Fluor 546 donkey anti-rabbit | Servicebio | GB21303 |  |
| rabbit anti-Oxytocin-neurophysin 1 | abcam | EPR20973 |  |
| **Bacterial and viruss trains** |  |  |  |
| rAAV9-hSyn-OT1.8 | Brain case Co., Ltd. | Cat#BC-1119 |  |
| Raav-EF1a-DIO-NES-jRGECO1a | Brain case Co., Ltd. | Cat#BC-0212 |  |
| rAAV2/9-OXT-Cre-WPRE-hGH-pA | BrainVTACo.,Ltd. | Cat#PT-6086 |  |
| rAAV2/9-CAG-DIO-axon-jGCaMP7b | BrainVTACo.,Ltd. | Cat#PT-8161 |  |
| rAAV2/9-DIO-EF1a-hChR2 (H134R)-mCherry | BrainVTACo.,Ltd. | Cat#PT-3787 |  |
| rAAV2/9-DIO-EF1a- eNpHR3.0-mCherry | BrainVTACo.,Ltd. | Cat#PT-0007 |  |
| rAAV2/5- EF1a-DIO-tettoxicP2A-mcherry | BrainVTACo.,Ltd. | Cat#PT-2139 |  |
| rAAV-EF1a-DIO-GCaMp6m-WPRE-hGH polyA | BrainVTACo.,Ltd. | Cat#PT-0283 |  |
| rAAV2/9-DIO-Ef1a-mCherry | BrainVTACo.,Ltd. | Cat#PT-0115 |  |
| **Experimental models:Organisms/strains** | | |  |
| Mouse: C57BL/6J | Beijing Vital River Laboratory Animal Technology Co., Ltd. | SCXK：2022-0030 |  |
| Mouse: CCK-Cre (C57BL/6) | Beijing Vital River Laboratory Animal Technology Co., Ltd. | Gifted by Professor Zheman Xiao's research group | |

OT1.8 -1983bp:

“ATGGAGACAGACACACTCCTGCTATGGGTACTGCTGCTCTGGGTTCCAGGTTCCACTGGTGACACAAGTTTGTACAAAAAAGTTGGCACCACCGGTGAGGGTGCGTTTGCGGCTAACTGGAGCGCTGAGGCGGTCAACGGGAGCGCGGCGCCGCCGGGAACCGAGGGCAATCGCACTGCCGGGCCGCCACAGCGCAACGAGGCCCTGGCGCGGGTGGAGGTGGCCGTGCTGTGCCTCATCCTGTTCCTGGCGCTGAGCGGCAACGCGTGCGTGCTGCTAGCGCTGCGCACCACGCGCCACAAGCACTCGCGCCTCTTCTTCTTCATGAAGCACCTGAGCATAGCCGACCTGGTAGTGGCGGTGTTCCAGGTGCTGCCGCAGCTTCTGTGGGACATCACGGGGCGCTTCTACGGGCCCGACCTGCTGTGCCGCCTCGTCAAGTACCTGCAGGTTGTGGGCATGTTCGCGTCCACCTACCTGCTGCTGCTCATGTCGCTCGACCGCTGCCTGGCCATCTGCCAGCCGCTGCGCTCGCTGAGCCGCCGCACCGACCGCCTGGCGGTACTCGTCACATGGCTCGGCTGCCTGGTGGCCAGCGCGCCGCAGGTGCACATCTTCTCGCTGCGCGAGGTGGCCGACGGTGTCTTCGACTGCTGGGCCGTTTTCATTCAACCCTGGGGGCCCAAGGCCTACATCACGTGGATCACGCTCGCCGTCTACATTGTGCCCGTCATCGTGCTTGCCACCTGCTATGGCCTTATCAGCTTCAAGATCTGGCAGAATGCGCGGCTCGCTACGGCGGGGCCGCGCGACACCGACGCGCTGGACCTGGAGGAGGGAGGAAACGTCTATATCAAGGCCGACAAGCAGAAGAACGGCATCAAGGCGGATTTCACCATCCGCCACAACATCGAGGACGGCGGCGTGCAGCTCGCCTACCACTACCAGCAGAACACCCCCATCGGCGACGGCCCCGTGCTGCTGCCCGACAACCACTACCTGAGCGTGCAGTCCAAACTTTCGAAAGACCCCAACGAGAAGCGCGATCACATGGTCCTGCTGGAGTTCGTGACCGCCGCCGGGATCACTCTCGGCATGGACGAGCTGTACAAGGGCGGTACCGGAGGGAGCATGGTGAGAAAGGGCGAGGAGCTGTTCACCGGGGTGGTGCCCATCCTGGTCGAGCTGGACGGCGACGTAAACGGCCACAAGTTCAGCGTGTTGGGCGAGGGTGAGGGCGATGCCACCGAGGGCAAGCTGACCCTGAAGTTCATCTGCACCACCGGCAAGCTGCCCGTGCCCTGGCCCACCCTCGTGACCACCCTGACCTACGGCGTGCAGTGCTTCAGCCGCTACCCCGACCACATGAAGCAGCACGACTTCTTCAAGTCCGCCATGCCCGAAGGCTACATCCAGGAGCGCACCATCTTCTTCAAGGACGACGGCAACTACAAGACCCGCGCCGAGGTGAAGTTCGAGGGCGACACCCTGGTGAACCGCATCGAGCTGAAGGGCATCGACTTCAAGGAGGACGGCAACATCCTGGGGCACAAGCTGGAGTACAACACCGGAGCAGCAGCACGCTGGCGCGGGCGGCAGAACAAGCTCATCTCTAAGGCCAAGATCCGCACGGTCAAGATGACCTTCATCGTCGTGCTGGCCTTCATCGTGTGCTGGACGCCATTCTTTTTCGTGAGTATGTGGAGTGTCTGGGATGCCGATGCGCCCAAGCACGCCTCACCTTTCATCATCGCCATGCTCCTGGCCAGCCTCAACAGCTGCTGCAACCCCTGGATCTACATGCTCTTCACGGGCCACCTCTTCCAAGAACTTGTGCAGCGCTTCCTCTGCTGCTCATTCCGCCGCCTGAAAGGCAGCCGGCCTGGGGAGACAAGCGTCAGCAAAAAGAGCAACTCGTCTACCTTTGTCCTGAGCCAGTACAGCTCCAGCCAGAGAAGATGCTCGCAGCCATCCACGCTGTGA”
